# The novel B-cell epitope peptide vaccine, MAX449, exhibits significant anti-tumor efficacy and enhances the therapeutic effects of PD-1 antibodies on tumors by modulating the activity of PMN-MDSCs

**DOI:** 10.7150/thno.122439

**Published:** 2026-01-14

**Authors:** Hong Yang, Xiao Han, Boshao Deng, Yunpei Zhao, Jing Zhao, Yufei Wu, Guokang Liu, Shiyu Zeng, Siyi Wang, Zhejuan Shen, Lulu Wang, Zihan Sun, Wenping Lu, Yuzhang Wu, Jian Chen

**Affiliations:** 1 Department of Immunology, Army Medical University (Third Military Medical University), Chongqing 400038, China.; 2 Department of Pharmacy, Southwest Hospital, Third Military Medical University, Chongqing, China.; 3 Biomedical Analysis Center, Army Medical University (Third Military Medical University), Chongqing 400038, China.; 4 Department of breast and thyroid Guiqian international general hospital, Guiyang 550000, China.; 5 Faculty of Hepato-Pancreato-Biliary Surgery, First Medical Center, Chinese PLA General Hospital, Beijing 100853, China.

**Keywords:** C5a-C5aR pathway, B-cell epitope peptide vaccine, MAX449, tumor immunotherapy, PD-1 antibody

## Abstract

**Rationale:** Evidence accumulating across experimental studies and clinical settings supports a central role for the C5a-C5aR signaling axis in promoting tumor progression and immune evasion. Nevertheless, whether a vaccination approach targeting C5a can elicit robust anti-tumor immune responses and suppress tumor growth has not yet been investigated. This research aimed to develop an efficient B-cell peptide epitope vaccine targeting the C5a-C5aR pathway for cancer therapy.

**Methods:** Chimeric C5a B-cell peptide epitope vaccines were synthesized using high-performance liquid chromatography (HPLC), and C5a antibodies titers were determined using enzyme-linked immunosorbent assay (ELISA). Multiple mouse tumor models were employed to evaluate the vaccine's efficacy. The mechanisms of MAX449 were assessed through in vitro and in vivo approaches, incorporating single-cell RNA sequencing (scRNA-seq), flow cytometry, western blotting, real-time quantitative PCR, transwell migration assays and ELISA.

**Results:** The vaccine MAX449 could induce high titer of C5a antibodies and effectively suppress tumor growth in multiple mouse models. Furthermore, MAX449 significantly boosted the effectiveness of anti-PD1 therapy. It not only inhibited the migration of polymorphonuclear myeloid-derived suppressor cells (PMN-MDSCs) to the tumor microenvironment through downregulating CCRL2 expression via the NF-κB signaling pathway but also reduced the immunosuppressive function of PMN-MDSCs by decreasing IL-1β production through the same pathway. Following vaccine administration, a significant expansion of anti-tumor CD8⁺ T cells was observed. Most importantly, the vaccine proved to augment the antitumor efficacy of programmed death-1 (PD-1) antibodies in cold and hot tumor mouse models.

**Conclusions:** This research demonstrated that MAX449 induced C5a antibodies, which block C5a-C5aR pathway in PMN-MDSCs, suppression of their migratory and immunosuppressive functions, and consequent antitumor activity. Meanwhile, MAX449 boosted the therapeutic efficacy of PD-1 antibody in hot and cold tumor model mice. This study provides compelling evidence supporting the clinical evaluation of MAX449 as an innovative therapeutic approach for cancer.

## Introduction

Although substantial progress has been made in early detection and therapeutic approaches, breast cancer continues to be the most common malignancy in women and ranks as the second leading cause of cancer-related mortality 1. Conventional treatment options include chemotherapy 2, radiotherapy 3 and surgery. However, these therapies are hardly cell-specific most of the time. Immunotherapy is a method by either actively or passively stimulating an immune response specifically directed at tumor, thereby facilitating the suppression and elimination of tumor cells 4. This therapeutic approach harnesses and enhances the immune response, allowing for targeted attacks on malignant cells 5,6. As a result, immunotherapy has emerged as a powerful clinical approach for cancer therapy, with an increasing number of drug approvals and a multitude of therapies undergoing clinical and preclinical development.

Among the diverse immunotherapeutic approaches, monoclonal antibodies (mAbs) targeting the PD-1 pathway by blocking its interaction with ligands have yielded the most robust clinical outcomes in breast cancer. The U.S. Food and Drug Administration (FDA) has granted approval to multiple PD-1-targeting monoclonal antibodies for clinical use in breast cancer 7. By antagonizing PD-1 signaling, these agents relieve T-cell suppression, thereby restoring anti-tumor immune responses. However, blockade of PD-1 alone is insufficient to overcome all mechanisms of therapeutic resistance, and a subset of patients exhibits limited clinical benefit from anti-PD-1 treatment. To address this limitation, therapeutic combinations aimed at simultaneously modulating multiple immune pathways have been proposed to enhance the efficacy of PD-1-directed interventions 8. Mechanistically, effective immunotherapeutic synergy relies on both the reactivation of effector T cells and the concurrent depletion of immunosuppressive populations, including myeloid-derived suppressor cells (MDSC) or regulatory T cells (Treg). Notably, PD-1/PD-L1 inhibition fails to adequately relieve T-cell suppression within the tumor microenvironment, a condition that is often reinforced by the accumulation of MDSCs 9 or Tregs 10. Accumulating evidence reveal that certain breast and colorectal cancer patients do not experience enduring clinical benefits from anti-PD-1 immunotherapy due to the infiltration of PMN-MDSCs within tumor microenvironment 11. Additionally, a subset of melanoma patients receiving PD-1 blockade experiences hyper progression, a phenomenon associated with enhanced accumulation of PMN-MDSCs in the lungs 12. Thus, strategies aimed at reversing tumor-associated immunosuppression may provide a pivotal means to overcome resistance to immunotherapy 13.

C5a, an anaphylatoxin generated as a biologically active proteolytic product during complement activation, plays a significant role in tumor progression by fostering an immunosuppressive tumor microenvironment in which myeloid-derived suppressor cells (MDSCs) are key contributors 14-16. The complement system, an essential arm of innate immunity, culminates in the proteolytic activation of the key components C3 and C5, leading to the generation of the anaphylatoxins C3a and C5a 17,18. While C5a is primarily recognized as a chemoattractant for proinflammatory leukocytes, emerging evidence reveal its ability to promote tumor progression, leading to a growing interest in complement blockade as a potential cancer treatment strategy 19-24. C5a further influences tumor progression by shaping an immunosuppressive tumor microenvironment involving MDSCs 24,25. Increasing evidence from both preclinical models and clinical trials indicates that the C5a-C5aR axis plays a key role in enabling tumors to evade immune surveillance and sustain growth, and that disruption of C5a-C5aR interactions has shown promising antitumor efficacy 21. Collectively, these findings underscore the essential involvement of C5a-C5aR signaling in antitumor immune regulation and suggest this axis as a viable therapeutic intervention within checkpoint inhibitor strategies.

Monoclonal antibodies targeting C5a and its receptor C5aR have demonstrated enhanced anti-tumor effects in cancer patients 26,27. Several Food and Drug Administration (FDA) approved C5a-C5aR mAbs, including GOHIBIC™ (Vilobelimab), avdoralimab (IPH 5401) 26, and 607 TJ210 (MOR210) 27, are currently undergoing clinical trials as checkpoint inhibitors for cancer treatment. Monoclonal antibodies targeting C5a or C5aR have demonstrated encouraging efficacy in pre-clinical and initial clinical settings; however, their successful clinical implementation is hampered by low overall response rates, treatment-related toxicities, development of resistance, and high manufacturing costs 28. Therefore, there is an urgent demand for novel therapeutic strategies. B-cell epitope-based chimeric peptides containing a broadly reactive T-cell epitope are utilized in active immunotherapy, eliciting a polyclonal antibody response 28. This approach offers a safer and more cost-effective therapeutic advantage compared to monoclonal antibodies 28.

In the present study, two novel C5a-targeted chimeric B-cell peptide epitope vaccines were developed, named MAX449 and MAX450, aimed at inducing polyclonal antibodies against the C5a-C5aR signaling pathway. To validate the efficacy of the most promising C5a B-cell epitope vaccine, we utilized multiple tumor models, including (i) BALB/c mice bearing 4T1 breast tumors and CT26 colon carcinoma, and (ii) C57BL/6 mice implanted with B16F10 melanoma to assess the impact of the C5a B-cell peptide epitope vaccines. Results demonstrated that MAX449 effectively suppressed tumor progression across multiple models. Notably, MAX449 markedly augmented the therapeutic efficacy of PD-1 antibody in multiple mouse models. Furthermore, we discovered that MAX449 exhibited its antitumor activity by inhibiting the migration of PMN-MDSCs to the tumor microenvironment, mediated by NF-κB-dependent downregulation of the atypical chemerin receptor CCRL2. Additionally, MAX449 suppressed the immunosuppressive functions of PMN-MDSCs by reducing IL-1β production by the NF-kB signaling pathway. Collectively, this newly constructed C5a B-cell epitope vaccine presents a promising and enhanced treatment option for patients exhibiting poor responses to anti-PD-1 treatment.

## Materials and Methods

### Peptide synthesis

Based on the C5a vaccines (KLH-C-KDMQLGR) 29, we modified its helper T cell epitope. Two chimeric C5a vaccines were generated by linking the C5a B-cell epitope sequence (KDMQLGR) to either the measles virus fusion peptide MVF (aa 288-302, KLLSLIKGVIVHRLEGVE) or the tetanus toxoid-derived peptide TT3 (aa 947-967, FNNFTVSFWLRVPKVSASHL) using a four-residue GPSL spacer 28. All C5a B-cell epitope peptide was synthesized and purified using solid phase peptide synthesis and high-performance liquid chromatography (HPLC) to ensure purity and quality, purchased from PeptideValley (Nanjing, China). Three-dimensional modeling of peptide secondary structures was performed with PyMOL software (version 2.4.0; Schrödinger, New York, NY, USA) according to the PyMOL User's Manual: https://pymol.org.

### Animals: C57BL/6 and BALB/c mice

Housing and experimental use of C57BL/6 and BALB/c mice complied with the biosafety regulations of Army Medical University. Transgenic PyMT (MMTV-PyMT) mice were commercially obtained (Shanghai Model Organisms Center, Inc (stock no. 6688265. SH), and breeding was maintained on C57BL/6 and PyMT (MMTV-PyMT) backgrounds. All in vivo experiments were approved by the Institutional Animal Care and Use Committees of Army Medical University (AMUWEC20230445).

### Animal immunization

Immunization followed a 4-times interval strategy 29. One milligram of peptide (SEQ ID NO.7, MAX449, MAX450, and MVF) was dissolved in 1× PBS (Servicebio, Cat: G0002-15) and subsequently adsorbed onto 0.2% Alhydrogel (Brenntag Biosector, Denmark). Each mouse was subcutaneously immunized with 100 μL on days 4, 6, 8, and 10 after tumor establishment. Mice were administered 250 μg of Anti-CD20 (Abinvivo, Cat: B7431) on day 3 via intraperitoneal injection 30, followed by a tail intravenous injection of 1 mg·kg^-1^ of C5a receptor antagonists (C5aRA) on days 4 and 6 31. Additionally, intraperitoneal treatment with anti-Ly6G (200 μg; Leinco Technologies, Cat: L280) was administered starting on day 3 11, and another intraperitoneal injection of 20 mg·kg^-1^ of Bay11-7082 was given on day 4 32.

### Antibody purification

Serum antibodies from immunized animals were isolated with a Protein A/G-based purification kit (biosharp, Cat: BL1264A). Refer to the manual for complete information.

### Cell lines

The murine breast cancer cell line 4T1, melanoma cell line B16F10, and colon carcinoma cell line CT26 were obtained from Procell Life Science and Technology. 4T1 cells were routinely maintained in high-glucose Dulbecco's Modified Eagle's Medium (DMEM; Gibco, Life Technologies, USA), whereas CT26 and B16F10 cells were cultured in RPMI-1640 medium (Gibco, Life Technologies, USA). All media were supplemented with 10% fetal bovine serum (FBS) and 100 U/mL penicillin-streptomycin, and cells were incubated at 37 °C in a humidified atmosphere containing 5% CO₂.

### Tumor implantation and assessment

BALB/c mice bearing CT26 (♂) or 4T1 (♀) tumors and C57BL/6 mice bearing B16F10 (♂) tumors were used to generate tumor models. Six- to eight-week-old mice received subcutaneous inoculation of 3 × 10⁵ tumor cells. Tumor growth was monitored by caliper measurement of length and width, and volumes were calculated as (length × width²)/2 33. Following sacrifice, tumors were excised and weighed. All animal housing and experimental procedures complied with institutional biosafety standards and were conducted with approval from the Institutional Animal Care and Use Committees of Army Medical University (AMUWEC20230445).

### Enzyme-linked immunosorbent assay (ELISA)

The immunogenic response was determined by ELISA following standard laboratory procedures 29. Plates were coated overnight with 1 μM C5a peptide (SEQ ID NO:7) and subsequently blocked with 1% BSA in PBS for 1 h at 37 °C. Serum samples (1:2000 dilution) were incubated for 3 h at 37 °C, after which HRP-labeled secondary antibody (Beyotime Biotechnology, Cat: A0216) was applied for 40 min. Signal development was performed using TMB substrate for 10 min at 37 °C and stopped by addition of stop solution. Optical density was recorded at 450 nm, and antibody titers were calculated as the dilution yielding half-maximal absorbance (ODmax/2) 29.

Levels of IL-1β in PMN-MDSC culture supernatants were quantified using a high-sensitivity Mouse IL-1β ELISA kit (ABclonal, Cat: RK04878) following the manufacturer's guidelines.

### Western blotting

Total cellular proteins were extracted by incubating cells on ice for 30 min in T-PER® lysis buffer (Thermo Scientific, XI355180) containing protease (1%; ABclonal, Cat: RM02916) and phosphatase inhibitors (1%; Servicebio, Cat: G2007-1ML). Protein concentrations were quantified using a NanoPhotometer® N50 (Implen), followed by denaturation in loading buffer (Beyotime Biotechnology, Cat: P0015L). Equivalent protein amounts were resolved by SDS-PAGE and transferred to PVDF membranes (immobilon®-p, Ref: IPVH00010). After blocking with Protein-Free Blocking Buffer for 1 h at room temperature, membranes were incubated with primary antibodies overnight at 4 °C and then with HRP-linked secondary antibodies (Beyotime Biotechnology, Cat: A0208) for 1 h at room temperature. Chemiluminescent signals were visualized using a Chemiluminescence Imaging System (Servicebio, Cat: SCG-W2000) with WesternBright ECL HRP substrate (Advanta), and band intensities were quantified using ImageJ software. The following primary antibodies were used: Alpha Tubulin (proteintech, Cat:11224-1-AP), Phospho-NF-κB p65 (proteintech, Cat: 82335-1-RR), CCRL2 (Aifang biological, Cat: AF04218), IL-1β (ABclonal, Cat: A16288), NLRP3 (ABclonal, Cat: A5652), Caspase-1 (ABclonal, Cat: A0964).

### Isolation of cells and flow cytometry

Single-cell suspensions were prepared from tumor tissues and selected organs of 4T1 tumor-bearing mice. For tumor analysis, excised tumors were finely minced and processed using a Mouse Tumor Dissociation Kit (Miltenyi, Cat: 130-096-730), followed by enzymatic digestion for approximately 42 min using a gentleMACS™ Octo Dissociator with Heaters (Miltenyi, Cat: 130-096-427). The resulting cell suspensions were filtered through 70 μm strainers (Beyotime Biotechnology, Cat: FSTR070) to remove debris and aggregates.

For bone marrow analysis, femurs were isolated, cleared of surrounding tissues, and flushed with sterile buffer using a 1 mL syringe, after which the cell suspension was passed through a 70 μm filter. Splenocyte suspensions were obtained by mechanically dissociating spleens through 70 μm cell strainers, followed by washing with FACS buffer (1× PBS containing 1% FBS).

Cells from all tissues were resuspended in FACS buffer, centrifuged at 300 g for 5 min at 4 °C, and subjected to red blood cell lysis using erythrocyte lysis buffer (Biosharp, Cat: BL503A) for 5 min at 4 °C. After washing, cells were incubated with fluorochrome-conjugated antibodies at 4 °C for 30 min. Following additional washing steps, cells were resuspended in FACS buffer and analyzed using a BD FACSymphony™ A1 flow cytometer (BD Biosciences).

To evaluate the frequency and functional status of total MDSCs and their subsets (PMN-MDSCs and M-MDSCs), cells were stained with antibodies against Live/Dead dye (BioLegend, Cat: 423105), CD45 (Cat: 103132), CD11b (Cat: 101263), Gr-1 (Cat: 108406), Ly6C (Cat: 128037), Ly6G (Cat: 127639), CCRL2 (Cat: 114007), and intracellular IL-1β (eBioscience™, Cat: 11-7114-82).

For CD8⁺ T-cell analysis, cells were surface-stained with antibodies against CD45 (Cat: 103132), CD3 (Cat: 100306), CD8 (Cat: 100752), and PD-1 (Cat: 135205), followed by intracellular staining for IFN-γ (BioLegend, Cat: 505826).

### Isolation of immune cells from tumor and cell sorting

Thirteen days after 4T1 tumor implantation, mice were euthanized and tumors were minced into small fragments, which were treated with a tumor Dissociation Kit for 42 min at atgentleMACS™ Octo Dissociator with Heaters. Single-cell suspensions were then labeled with Live/Die and CD45 (Biolegend, Cat: 147711) and subsequently sorted using a FACS Aria III cell sorter (BD Biosciences).

### Single-cell RNA sequencing analysis

Single-cell RNA sequencing (scRNAseq) data processing and analysis were conducted by NovelBio Bio-Pharm Technology Co., Ltd. with NovelBrain Cloud Analysis Platform. Raw sequencing data in FASTQ format were initially processed to remove adaptor sequences and low-quality reads using default filtering parameters, yielding high-quality clean data. UMI-tools were employed to identify valid cell barcodes and generate a barcode whitelist for downstream single-cell transcriptome analysis. Clean UMI-based reads were aligned to the mouse reference genome (Ensembl version 100) using STARmapping with customized parameters derived from the standard UMI-tools pipeline, allowing generation of UMI count matrices for each sample. Cells containing more than 200 expressied genes and exhibiting a mitochondrial UMI proportion below 20% were retained for further analysis, and mitochondrial genes were excluded from the final expression matrix. Following removal of low-quality cells and contaminating non-immune lineages, a total of 7,949 immune cells were included in the analysis (Ctrl group: 4,304 cells; MAX449 group: 3,645 cells). Data normalization, scaling, and regression were performed using the Seurat package (version 3.1.4; https://satijalab.org/seurat/), accounting for UMI counts and mitochondrial gene content. Principal component analysis (PCA) was conducted based on the top 2,000 highly variable genes, and the first 10 principal components were subsequently used for Uniform Manifold Approximation and Projection (UMAP) visualization.

Unsupervised clustering was performed using a graph-based approach with a resolution parameter set to 0.8, based on the first 10 principal components derived from PCA. Marker genes for each cluster were identified using the FindAllMarkers function with the Wilcoxon rank-sum test, applying the following thresholds: lnFC > 0.25, P value < 0.05, and minimum expression in more than 10% of cells (min.pct > 0.1). For refined cell-type annotation, clusters corresponding to the same cell lineage were further subsetted and subjected to secondary UMAP visualization, graph-based reclustering, and marker gene analysis. Differentially expressed genes were identified using DESeq2 with cutoffs of |log₂ fold change| > 0.25 and P < 0.05, and the resulting gene expression patterns were visualized using heatmaps. Functional enrichment analyses were conducted to identify significantly altered biological pathways based on the Kyoto Encyclopedia of Genes and Genomes (KEGG) and Gene Ontology (GO) databases. Fisher's exact test was applied to assess pathway enrichment, and statistical significance was determined according to both P values and false discovery rate (FDR) thresholds.

Differential gene expression analysis was conducted for all cell populations within the filtered and integrated datasets using the FindMarkers function (Wilcoxon rank-sum test) implemented in the Seurat package. For each experimental group, cell type-specific gene expression matrices were generated, and the top 20 genes with P values < 0.01 were selected as representative markers for each cell population. Heatmaps were constructed using the ComplexHeatmap package, with the selected marker genes annotated alongside the plots. To explore the biological functions associated with individual cell clusters, Gene Ontology (GO) enrichment analysis was performed on cluster-specific genes using the clusterProfiler package. The top 15 significantly enriched pathways (P < 0.05) were selected and visualized as bar plots using the ggplot2 package.

Single-cell trajectory inference was performed using Monocle2 (http://cole-trapnell-lab.github.io/monocle-release) with the DDRTree algorithm and default parameters. Prior to trajectory construction, marker genes identified from Seurat-based clustering and raw expression count matrices from quality-filtered cells were selected as input for Monocle analysis. Pseudotime ordering was subsequently applied to reconstruct cell state transitions, and branch expression analysis modeling (BEAM) was used to identify genes associated with lineage bifurcation and cell fate decisions. For annotation of Monocle-defined states, we curated and integrated previously reported marker gene sets characteristic of MDSCs and neutrophil populations, including neutrophil progenitors, mature neutrophils, transitional PMN-MDSCs, and terminal PMN-MDSCs, as described in published studies 34. Cell functional scores derived from predefined gene sets were calculated using the AddModuleScore function.

### Isolation of PMN-MDSCs

Isolation of PMN-MDSCs was carried out based on the method established by Chaoxiong Wang et al 35. Bone marrow-derived single-cell suspensions were first subjected to red blood cell lysis, followed by enrichment of PMN-MDSCs using a commercial Mouse Bone Marrow Neutrophil Isolation Solution Kit (Solarbio, Cat: P8550) according to the supplier's protocol.

### Transwell migration assays

Cell migration was assessed using Transwell insert chambers with 8-μm pore membranes (Corning). PMN-MDSCs were isolated from mice within the same experimental group and pooled at equal numbers. Cells were preincubated with or without C5aRA/MAX449-induced antibodies for 1 h, after which 6 × 10⁵ PMN-MDSCs were plated into the upper chamber in 100 μL of medium containing 0.5% FBS. The lower chambers were filled with medium supplemented with 10% FBS as the control condition, or with C5a-treated conditioned medium containing 10% FBS for experimental conditions. After 6 h of incubation, cells that had migrated through the membrane were collected, and 10 μL of the cell suspension was counted under a microscope.

### RNA extraction and qRT-PCR

Total RNA was isolated from cells using the RNeasy™ Animal RNA Isolation Kit with Spin Columns (Beyotime Biotechnology, Cat: R0026) and subsequently reverse transcribed into cDNA with the ABScript Neo RT Master Mix for qPCR with gDNA removal (ABclonal, Cat: RK20433). Quantitative real-time PCR was carried out using 2× Universal SYBR Green Fast qPCR Mix (ABclonal, Cat: RK21203) on a Roche LightCycler® 480 system. Each sample was analyzed in triplicate with a final reaction volume of 10 μL. TBP served as the internal control, and relative gene expression levels were calculated using the 2^-ΔΔCt method.

Primer sequences are listed:

CCRL2 Forward (5'→3'): TGTGTTTCCTGCTTCCCCTG,

CCRL2 Reverse (5'→3'): CGAGGAGTGGAGTCCGACAA;

TBP Forward (5'→3'): CCCCACAACTCTTCCATTCT,

TBP Reverse (5'→3'): TACTGGGGATAGTGAGGACG.

### Statistical analysis

All statistical analyses were performed using GraphPad Prism software (version 8.4.0). Comparisons between two groups were conducted using a two-tailed unpaired Student's t-test with a 95% confidence interval, while multiple-group comparisons were analyzed by one-way analysis of variance (ANOVA). Tumor growth differences were specifically evaluated using one-way ANOVA. Statistical significance was defined as follows: ****P < 0.0001, ***P < 0.001, **P < 0.01, *P < 0.05, and ns indicating no significance (*P* > 0.05). Data are presented as Mean ± s.e.m, unless otherwise stated.

## Results

### Newly modified C5a vaccines were developed and exhibited remarkably high immunogenicity

Previous studies have shown that blocking C5a-C5aR pathway could inhibit tumor growth in various mouse models 13,19,36. Current drug development targeting this pathway primarily focuses on monoclonal antibody therapies such as GOHIBIC™ (Vilobelimab), avdoralimab (IPH 5401), and 607 TJ210 (MOR210). However, these therapies face significant limitations, including low response rates, toxicity, development of resistance, and high costs 28. Therefore, novel immune therapies targeting C5a-C5aR pathway are urgently needed to enhance treatment efficacy and duration of response.

**Figure 1:**
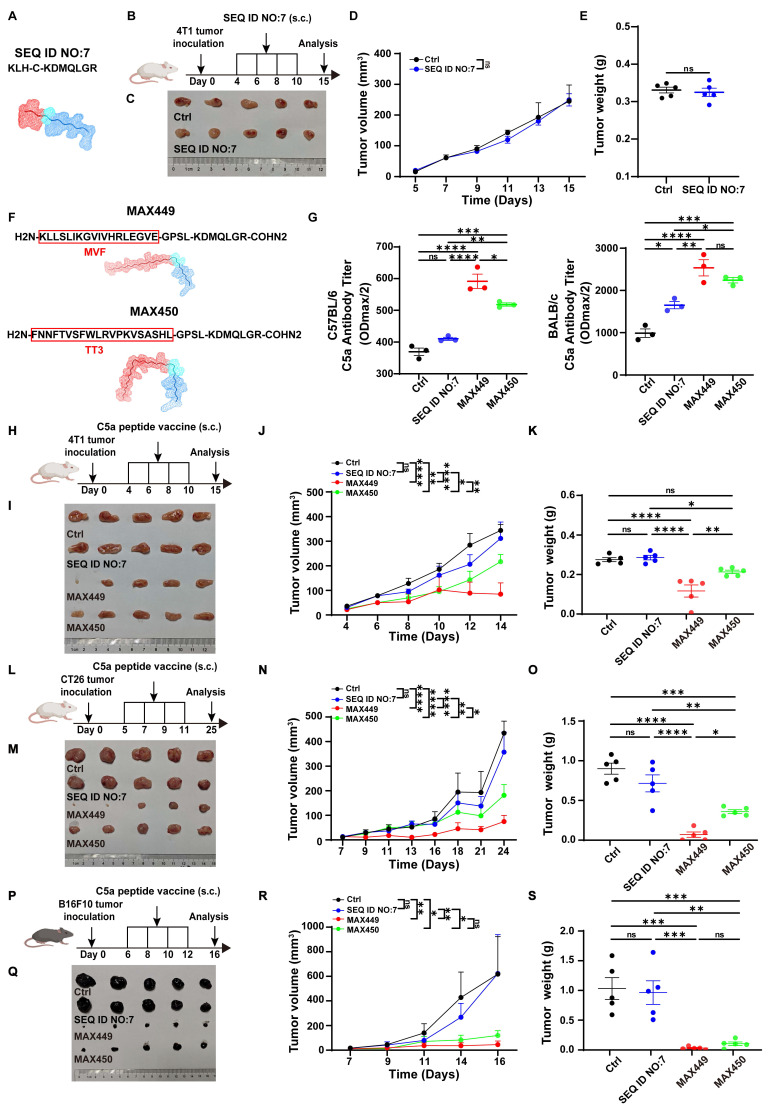
** C5a vaccines MAX449 had high immunogenicity and exhibited excellent anti-tumor effects across multiple mouse models.** (A) Amino acid sequences and secondary structure of SEQ ID NO.7 (CN103269711A)) peptide. (B) Experimental design of SEQ ID NO.7 vaccines on 4T1 tumor transplantation. One milligram of peptide was dissolved in 1 mL PBS, and each mouse was subcutaneously immunized with 100 μL on day 4, 6, 8, and 10 post-implantations (n = 5 mice per group). (C) On day 15 after the start of inoculation, the 4T1 tumors were collected. Differences in the overall growth curves of mean tumor volume (D) and tumor weight (E) were evaluated using *t*-tests. (F) Amino acid sequences and secondary structure of MAX449 and MAX 450 peptide. (G) Serum antibody titters in C57BL/6J and BALB/c mice at 8 days after prime first injection. (H) Experimental design of C5a peptide vaccines on 4T1 tumor transplantation. One milligram of peptide was dissolved in 1 mL PBS, and each mouse was subcutaneously immunized with 100 μL on day 4, 6, 8, and 10 post-implantations (n = 5 mice per group). (I) On day 15 after the start of inoculation, the 4T1 tumors were collected. One-way ANOVA was applied to evaluate differences across the complete growth curves of mean tumor volume (J) and tumor weight (K). (L) Experimental design of C5a peptide vaccines on CT26 tumor transplantation. One milligram of peptide was dissolved in 1 mL PBS, and each mouse was subcutaneously immunized with 100 μL on day 5, 7, 9, and 11 post-implantations (n = 5 mice per group). (M) On day 25 after the start of inoculation, the tumors were collected. One-way ANOVA was employed to assess differences in the complete growth curves of mean tumor volume (N) and tumor weight (O). (P) Experimental design of C5a peptide vaccines on B16F10 tumor transplantation. One milligram of peptide was dissolved in 1 mL PBS, and each mouse was subcutaneously immunized with 100 μL on day 6, 8, 10, and 12 post-implantations (n = 5 mice per group). (Q) On day 16 after the start of inoculation, the tumors were collected. One-way ANOVA was applied to examine differences across the complete growth curves of mean tumor volume (R) and tumor weight (S). The results are expressed as the Mean ± s.e.m. *P* values: **** < 0.0001, *** < 0.001, ** < 0.01, * < 0.05, ns > 0.05.

The B cell peptide vaccine presents multiple advantages, including sustained anti-tumor effects, rapid and scalable synthesis, favorable safety profiles with minimal toxicity, and low production costs, making it a promising strategy in cancer therapy area 28,37. However, there have been no reports of B cell peptide vaccines specifically targeting the C5a-C5aR pathway for tumor therapy. Consequently, we hypothesize that constructing C5a B cell epitope peptide vaccines could exert excellent anti-tumor effects. In previous research, Christine Landlinger developed a series of C5a-peptide vaccines that successfully generated strong and specific immunogenic responses targeting the pro-inflammatory factor C5a. These C5a B cell epitope peptide vaccines significantly mitigated neuronal impairment and improved disease-related symptoms in an Alzheimer's disease mouse model 29,38. For the anti-tumor effect assessment, a C5a peptide vaccine composed of the 68-74 amino acid region of C5a (SEQ ID NO:7, CN103269711A) conjugated to keyhole limpet hemocyanin (KLH) was selected for anti-tumor effect test based on its capability to induce high-titer anti-C5a polyclonal antibodies in the Alzheimer's disease mouse model (Figure 1A,) 29,38. Considering the critical importance of suppressing the C5a-C5aR signaling axis in the treatment of Alzheimer's disease therapy, we propose that this vaccine peptide may serve as a potential treatment for tumors. However, its anti-tumor effects were evaluated in breast cancer transplantation models, and the results indicated that it was ineffective (Figure 1B-E).

We hypothesized that the C5a peptide (SEQ ID NO:7, CN103269711A) exhibited low immunogenicity and couldn't effectively induce enough C5a antibody for eliminating tumors. Therefore, our objective was to modify the existing peptide to generate one with enhanced immunogenicity and potential anti-tumor properties by incorporating helper T cell (Th) epitope sequences to augment the immunogenicity of cytotoxic T lymphocyte (CTL) epitopes. Two Th epitope sequences were chosen and synthesized: the measles virus-derived fusion peptide (MVF; amino acids 288-302, KLLSLIKGVIVHRLEGVE) and a tetanus toxoid-derived peptide (TT3; residues 947-967, FNNFTVSFWLRVPKVSASHL) 28. Ultimately, two previously unreported C5a B cell epitope peptides were generated, named as MAX449 and MAX450 (Figure 1F). To further evaluate the immunogenicity of these two vaccines, we measured antibody levels induced by the vaccines in mouse models. The results revealed that C57BL/6 and BALB/c mice immunized with MAX449 and MAX450 exhibited significantly higher antibody levels than those receiving the pre-modified C5a vaccine (SEQ ID NO:7, CN103269711A) (Figure 1G), indicating that the MAX449 and MAX450 peptides possess enhanced immunogenicity and may exhibit superior anti-tumor effects.

### MAX449 demonstrated impressive anti-tumor effects in various mouse models by blocking the C5a-C5aR pathway through B cell-derived C5a antibody

To assess the therapeutic potential of the two newly modified C5a vaccines, we established a 4T1 xenograft mouse model, which was recognized as a typical cold tumor.

The tumor-bearing mice received treatment with C5a vaccines at early stages (Days 4, 6, 8, and 10) following tumor inoculation (Figure 1H). Among the groups, MAX449 exhibited the most significant anti-tumor activity, effectively restraining tumor growth in the mouse models. Conversely, the treatment with MAX450 resulted in a comparatively weaker therapeutic effect (Figure 1I-K). To further confirm the antitumor activity of the vaccine peptides, their therapeutic effects were assessed in another cold tumor model, the CT26 transplant tumor mouse model. As shown in Figure 1L-O, MAX449 demonstrated markedly superior anti-tumor activity in the CT26 xenograft model, whereas MAX450 exhibited a comparatively weaker therapeutic effect. These findings suggested that MAX449 consistently exhibited strong anti-tumor effects in cold tumor models. Additionally, we explored the anti-tumor efficacy of the vaccine peptides in the “B16F10 transplanted mouse tumor,” a hot tumor model, where MAX449 also displayed the best anti-tumor effect (Figure 1P-S). Collectively, these results confirm that MAX449 treatment significantly inhibits tumor growth across various mouse models.

**Figure 2:**
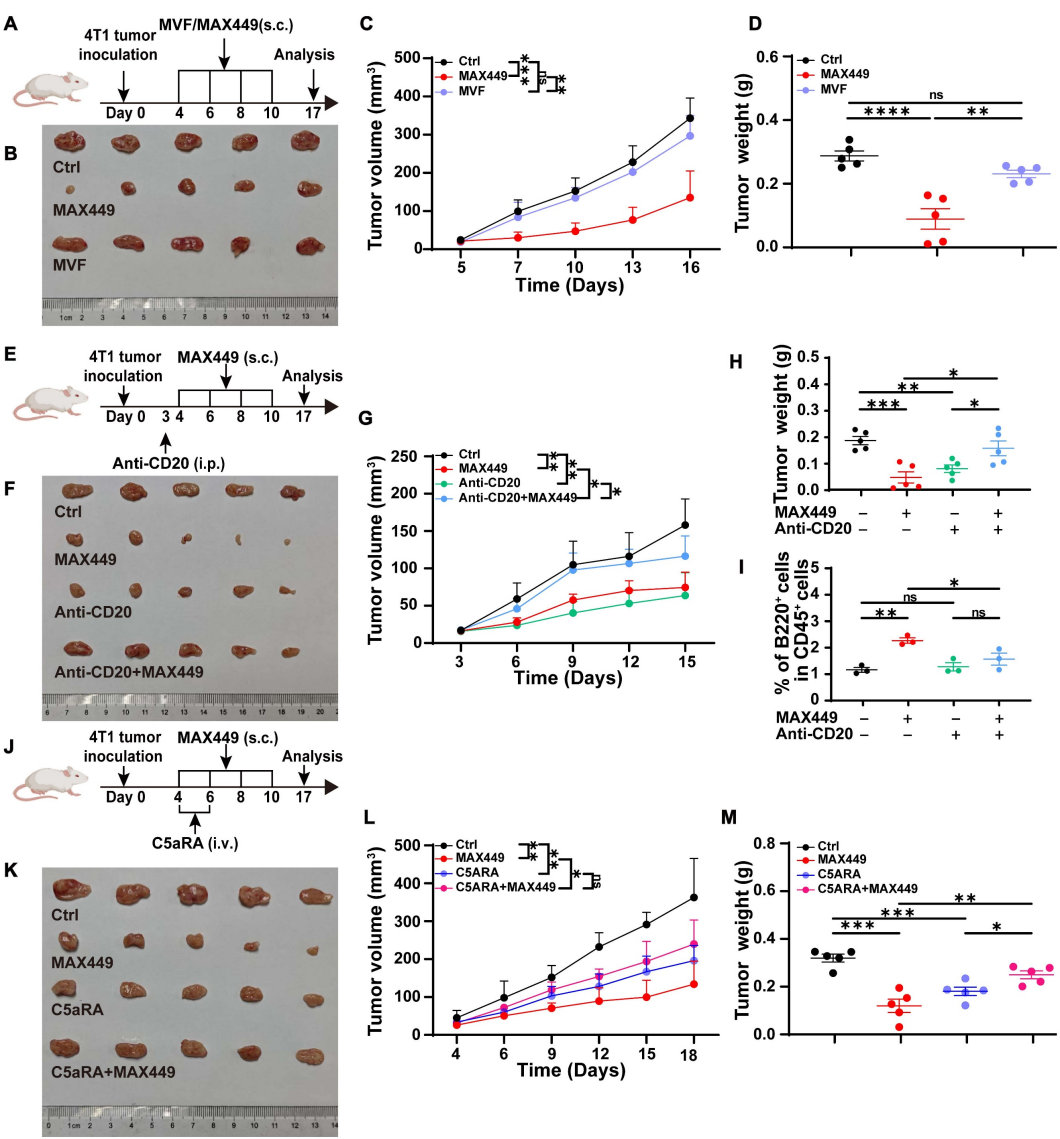
** MAX449 blocks C5a-C5aR signaling via B cell-derived anti-C5a antibody.** (A) Experimental design of MAX449 and MVF on 4T-1 tumor transplantation. One milligram of peptide MVF or MAX449 was dissolved in 1 mL PBS, and each mouse was subcutaneously immunized with 100 μL on day 4, 6, 8, and 10 post-implantations (n = 5 mice per group). (B) On day 17 after the start of inoculation, the 4T-1 tumors were collected. One-way ANOVA was applied to assess differences across the complete growth curves of mean tumor volume (C) and tumor weight (D). (E) Experimental design of Anti-CD20 and MAX449 on 4T-1 tumor transplantation. One milligram of peptide MAX449 was dissolved in 1 mL PBS, and each mouse was subcutaneously immunized with 100 μL on days 4, 6, 8, and 10 post-implantation (n = 5 mice per group). On day 3 after tumor implantation, mice received an intraperitoneal injection of 250 μg anti-CD20. (F) On day 17 after the start of inoculation, the tumors were collected. The full curves of average tumor volume (G) and tumor weight (H) were compared using a t-test. (I) Representative flow cytometry scatter plots of B cells on CD45^+^ cells. (J) Experimental design of MAX449 and C5aRA on 4T-1 tumor transplantation. One milligram of peptide MAX449 was dissolved in 1 mL PBS, and each mouse was subcutaneously immunized with 100 μL on day 4, 6, 8, and 10 post-implantation (n = 5 mice per group). Mice were administered a tail-vein injection of C5aRA at a dose of 1 mg·kg⁻¹ via intravenous route on day 4 and 6 post-implantations. (K) On day 17 after the start of inoculation, the 4T-1 tumors were collected. A t-test was performed to analyze the entire growth curves of mean tumor volume (L) and tumor weight (M). The data are presented as Mean ± s.e.m. *P* values: **** < 0.0001, *** < 0.001, ** < 0.01, * < 0.05, ns > 0.05.

MAX449 was designed by incorporating helper T cell (Th) epitope sequences MVF into the 68-74 amino acid region of C5a to enhance its immunogenicity. Exclude the possibility that the antitumor activity observed with MAX449 arises from the MVF peptide component, we immunized tumor-bearing mice with the MVF peptide alone (Figure 2A). The results presented in Figure 2B-D demonstrated that the MVF peptide alone had no anti-tumor effect. Subsequently, to determine whether the anti-tumor effect elicited by MAX449 relies on B cells, we administered an anti-CD20 antibody to deplete B cells in a mouse tumor transplant model. As depicted in Figure 2E-I, this B cell depletion abolished the anti-tumor effect of MAX449, indicating that its efficacy is B cell-dependent. Furthermore, to confirm that MAX449 exerts anti-tumor effects by inducing the production of C5a antibodies, which block the C5a-C5aR pathway, we treated tumor-bearing mice with C5aR receptor antagonist (C5aRA). Figure 2J-M demonstrated that tumor growth was significantly inhibited in both the C5aRA treatment group and the MAX449 group. However, tumor size did not differ significantly between mice treated with C5aRA alone and those receiving the combined C5aRA and MAX449 treatment, indicating that MAX449 mediated its anti-tumor effect by blocking the C5a-C5aR pathway.

### MAX449 modulated the tumor immune milieu

**Figure 3:**
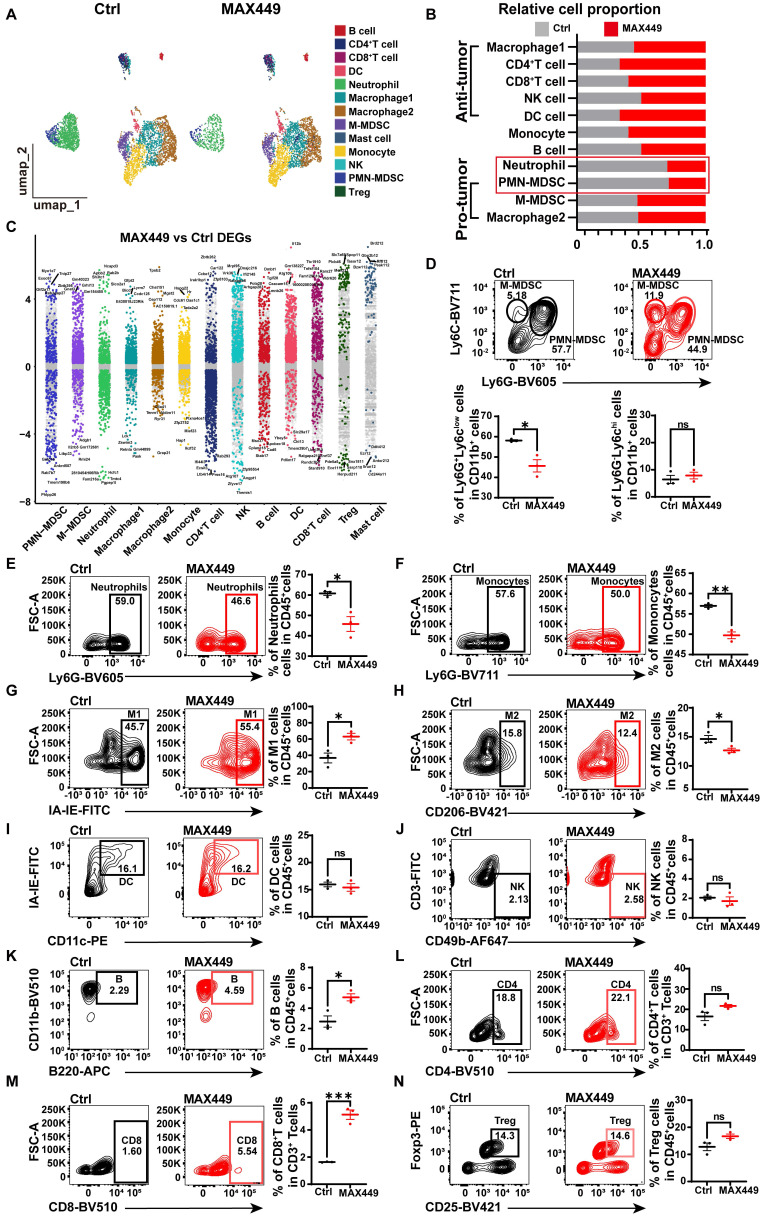
** ScRNA-seq and flow cytometry analysis revealed that MAX449 reversed tumor immunosuppressive microenvironment.** (A) Uniform Manifold Approximation and Projection (UMAP) plot in Ctrl and MAX449 groups. (B) Proportions of immune subpopulations in Ctrl and MAX449 groups. (C) Differentially expressed genes across distinct cell populations are shown. The y-axis indicates log₂-transformed expression fold changes, with genes exhibiting log₂FC > 0.5 highlighted in subgroup-specific colors. Key marker genes are labeled in the plot. Representative flow cytometry dot plots illustrate the distributions of PMN-MDSCs and M-MDSCs (D), neutrophils (E), monocytes (F), M1 macrophages (G), M2 macrophages (H), dendritic cells (I), NK cells (J), B cells (K), CD4⁺ T cells (L), CD8⁺ T cells (M), and regulatory T cells (Tregs) (N) in tumors from control and MAX449-treated mice (n = 3 per group). *P* values: **** < 0.0001, *** < 0.001, ** < 0.01, * < 0.05, ns > 0.05.

The tumor microenvironment (TME) plays a critical role in contributing to poor prognosis in cancer treatment, and its modulation offers a strategy to restore anti-tumor immunity. Previous research has shown that targeting the C5a-C5aR pathway can remodel the TME by influencing key immune cells such as MDSCs, macrophages and neutrophils 39. To further investigate the anti-tumor mechanism of MAX449, we analyzed tumor-infiltrated CD45^+^ immune cells using the NovelCyto/BD platform. Following rigorous quality control, transcriptional profiling was performed on 7,949 cells (Ctrl: 4,304 cells, MAX449: 3,645 cells), with cell populations resolved using nonlinear dimensional reduction and UMAP clustering, the CD45^+^ immune cells were classified into 13 distinct populations (Figure 3A). Relative to the control group, we detected a marked decrease in PMN-MDSCs and neutrophils, accompanied by an increased abundance of CD8^+^ T cells, CD4^+^ T cells and DC cells (Figure 3B). To explore the effects of MAX449 on immune regulation, we found that its administration significantly influenced the functional profiles of PMN-MDSCs, M-MDSCs and neutrophils (Figure 3C). Flow cytometric analysis further confirmed a pronounced increase in infiltrating M1 macrophages, CD8^+^ T cells and B cells and a decrease in PMN-MDSCs, Monocytes, M2 macrophages and neutrophils infiltration in tumors with MAX449 treatment (Figure 3D-N).

### MAX449 treatment shaped the transcriptional landscape of PMN-MDSC

**Figure 4:**
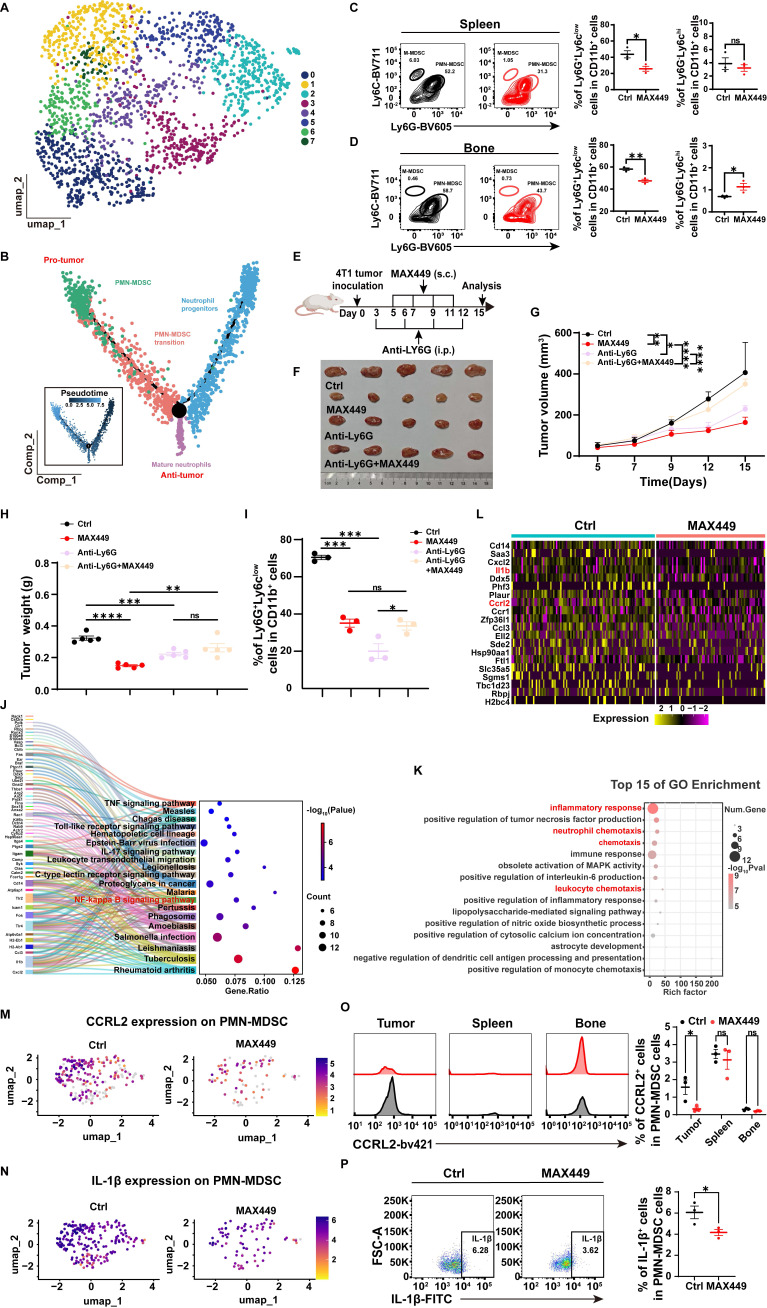
** MAX449 primarily modulated the tumor immunosuppressive microenvironment via PMN-MDSCs.** (A) UMAP view of Granulocyte cell clusters. (B) Trajectory analysis revealed granulocyte cell state transitions in two distinct groups. Representative flow cytometry scatter plots show PMN-MDSCs and M-MDSCs in the spleen (C) and bone marrow (D) from Ctrl and MAX449 groups (n = 3 mice per group). (E) Experimental design of MAX449 and Anti-Ly6G on 4T1 tumor transplantation. One milligram of peptide MAX449 was dissolved in 1 mL PBS, and each mouse was subcutaneously immunized with 100 μL on day 5, 7, 9, and 11 post-implantations. Mice were intraperitoneal injection of 200 μg of Anti-Ly6G on days 3, 6, 9, and 12 post-implantations (n = 5 mice per group). (F) On day 15 after the start of inoculation, the 4T1 tumor were collected. A t-test was applied to evaluate differences across the complete growth curves of mean tumor volume (G) and tumor weight (H). (I) Flow cytometry statistical graph of PMN-MDSCs in tumor tissues. KEGG enrichment analysis (J), GO enrichment analysis (K), and heatmap (L) of relative genes expressed on PMN-MDSC between Ctrl and MAX449 groups. Colors denote gene expression levels. Gene expression density of CCRL2 (M) and IL-1β (N) in PMN-MDSCs in tumor tissues between Ctrl and MAX449 groups. Color indicates log-normalized marker gene expression across cell stages (yellow to purple, low to high). Representative histograms and statistical analyses of CCRL2 (O) and IL-1β (P) expression in PMN-MDSCs from Ctrl and MAX449 tumor-bearing mice at day 13 after 4T1 inoculation. *P* values: **** < 0.0001, *** < 0.001, ** < 0.01, * < 0.05, ns >0.05.

As the results above demonstrated, in contrast to controls, MAX449 administration resulted in the most significant modulation of PMN-MDSC and neutrophil populations at both quantitative and transcriptional levels. Neutrophils and PMN-MDSCs, both belonging to the granulocyte family, share a common origin, follow similar differentiation pathways, and have related phenotypes 34. To evaluate the effects of MAX449 treatment on granulocytes, we performed clustering analyses to delineate distinct granulocyte subpopulations (Figure 4A).

Analysis of the granulocyte differentiation trajectory indicated that PMN-MDSCs primarily exert immunosuppressive effects within the tumor microenvironment, whereas mature neutrophils mainly perform anti-tumor functions. We speculate that MAX449 may exert its antitumor activity mainly through decreasing PMN-MDSCs abundance and inhibiting their immunosuppressive function. Furthermore, flow cytometry results indicated that following MAX449 treatment, the quantity of PMN-MDSCs significantly decreased not only in the tumor microenvironment (Figure 3D) but also in the bone marrow and spleen (Figure 4C-D), indicating that MAX449 may mediate its anti-tumor activity by suppressing PMN-MDSCs. To further confirm whether MAX449 mainly exerts anti-tumor effects by targeting PMN-MDSCs, we depleted MDSCs in mice using Anti-Ly6G and subsequently treated tumor-bearing mice with MAX449 (Figure 4E). As shown in Figures 4F-I, the anti-tumor effect of MAX449 diminished after MDSC depletion, indicating that MAX449 primarily exerts its anti-tumor effect by inhibiting PMN-MDSCs. To explore the mechanisms by which MAX449 regulates PMN-MDSC function, pathway analysis utilizing Kyoto Encyclopedia of Genes and Genomes (KEGG) enrichment identified several enriched pathways in PMN-MDSCs, including the TNF signaling pathway, IL-17 signaling pathway, and NF-κB signaling pathway (Figure 4J). Additionally, Gene Ontology (GO) enrichment analysis indicated that MAX449 treatment significantly decreased inflammatory response and chemotaxis-related activities in PMN-MDSCs (Figure 4K). Notably, differential gene expression analysis revealed downregulation of inflammation-related gene signatures (*Ccl3, Il1b,* and *Cxcl2*) in PMN-MDSCs, alongside a reduction in the expression of the migration-associated gene *Ccrl2* (Figure 4L-N). Flow cytometry experiments further confirmed a reduction in CCRL2 and IL-1β expression in PMN-MDSC cells within the tumor microenvironment of MAX449-treated mice (Figure 4O-P). Based on these findings, we proposed that MAX449 affect the recruitment of PMN-MDSCs and their immunosuppressive functions, thereby modulating the tumor immune milieu.

### MAX449 suppresses PMN-MDSC migration and immunosuppressive activity by downregulating CCRL2 and IL-1β via inhibition of the NF-κB signaling pathway

**Figure 5:**
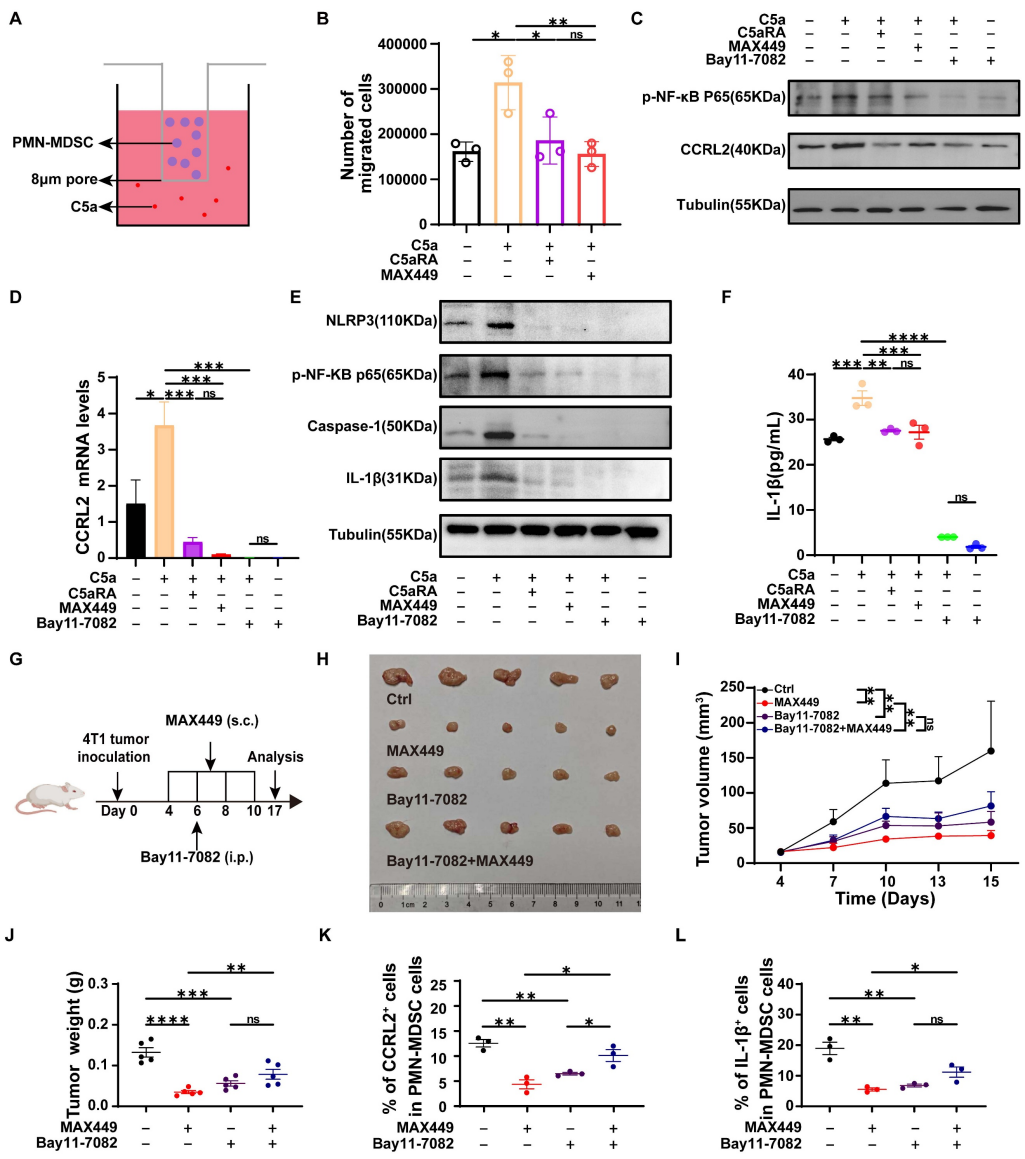
** MAX449 inhibits the migration and immunosuppressive function of PMN-MDSCs through suppressing NF-κB signaling pathway.** (A) Schematic illustration of the transwell migration assay. (B) Number of migrated cells of PMN-MDSCs in transwell (n = 3 per group). (C) Western blot analysis of CCRL2, *p*-NF-κB in PMN-MDSCs treated with indicated drugs (C5a, C5a+C5aRA, C5a+MAX449, C5a+Bay 11-7082, Bay 11-7082). (D) qRT-PCR analysis of CCRL2 level in PMN-MDSCs treated with indicated drugs (C5a, C5a+C5aRA, C5a+MAX449, C5a+Bay 11-7082, Bay 11-7082) (n = 3 per group). (E) Protein expression of *p*-NF-κB, NLRP3, caspase-1, and IL-1β in PMN-MDSCs was examined by Western blot after exposure to the indicated treatments (C5a, C5a+C5aRA, C5a+MAX449, C5a+Bay 11-7082, Bay 11-7082). (F) ELISA analysis of IL-1β level in PMN-MDSCs treated with indicated drugs (C5a, C5a+C5aRA, C5a+MAX449, C5a+Bay 11-7082, Bay 11-7082) (n = 3 per group). One-way ANOVA analysis was performed. All values are expressed as the Mean ± s.e.m. (G) Experimental design of Bay11-7082 and MAX449 on 4T-1 tumor transplantation. One milligram of peptide MAX449 was dissolved in 1 mL PBS, and each mouse was subcutaneously immunized with 100 μL on day 4, 6, 8, and 10 post-implantations. Mice were intraperitoneal injection of 20 mg·kg^-1^ of Bay11-7082 on day 6 post-implantations (n = 5 mice per group). (H) On day 17 after the start of inoculation, the tumors were collected. A t-test was employed to assess differences across the complete growth curves of mean tumor volume (I) and tumor weight (J). Representative statistics showing the expression of CCRL2 (K) and IL-1β (L) on PMN-MDSCs from Ctrl and MAX449 treated mice bearing 4T1 tumors at 17 days post inoculation. Results were shown as the Mean ± s.e.m. *P* values: **** < 0.0001, *** < 0.001, ** < 0.01, * < 0.05, ns >0.05.

Given the critical role of CCRL2 in directing cell migration to inflammatory sites 40. Its upregulation is essential for CXCR2-dependent recruitment of granulocytes 41. We hypothesized that MAX449 downregulated CCRL2 expression in PMN-MDSCs, thereby reducing their migration toward the tumor microenvironment. Transwell assays demonstrated that PMN-MDSCs migration increased following C5a stimulation, while blocking the C5a-C5aR pathway with C5aRA resulted in decreased migration. MAX449 treatment exhibited effects akin to those of C5aRA (Figure 5A-B). These findings suggested that MAX449 attenuated the recruitment of PMN-MDSCs to the tumor microenvironment through suppression of CCRL2 expression. Prior studies have demonstrated that NF-κB can directly associate with the *CCRL2* promoter to activate its transcriptionn 42. KEGG analysis demonstrated that genes associated with the NF-κB signaling pathway is enriched in PMN-MDSCs (Figure 4J). Therefore, we hypothesized that MAX449 downregulated CCRL2 expression in PMN-MDSCs by modulating of the NF-κB pathway. Western blot analysis revealed a significant increase in CCRL2 expression on PMN-MDSCs after C5a stimulation. However, this increase could be reversed by C5aRA, MAX449 induced antibodies, and the NF-κB inhibitor BAY11-7082 (Figure 5C). These findings were corroborated by qRT-PCR analysis (Figure 5D). Overall, our findings suggested that MAX449 decreased PMN-MDSCs migration by downregulating CCRL2 expression through inhibition of the NF-κB signaling pathway. In addition, IL-1β is a key regulator of the immunosuppressive activity of these cells 43-45. We hypothesized that MAX449 diminishes the immunosuppressive capacity of PMN-MDSCs by lowering IL-1β levels. Previous studies have demonstrated that NF-κB/NLRP3/caspase-1 regulated the expression of IL-1β 46. Thus, we posited that MAX449 reduced the immunosuppressive function of PMN-MDSCs by decreasing IL-1β secretion via the NF-κB pathway. To test this hypothesis, we assessed the phosphorylation of NF-κB components, NLRP3, Caspase-1, and IL-1β in PMN-MDSCs treated with or without C5a, C5aRA, and MAX449 induced antibodies. The data indicated that levels of *p*-NF-κB, NLRP3, Caspase-1, and IL-1β increased after C5a stimulation, while they decreased following treatment with MAX449 antibodies, C5aRA, and BAY11-7082 (NF-κB pathway inhibitor) (Figure 5E). These findings were further corroborated by ELISA assays (Figure 5F). In conclusion, the results suggested that MAX449 inhibited the migration and immunosuppressive function of PMN-MDSCs by downregulating CCRL2 and IL-1β expression via the NF-κB signaling pathway.

To determine whether suppression of the NF-κB/CCRL2 and NF-κB/IL-1β axes by MAX449 contributes to its antitumor activity, 4T-1 xenograft mice were administered with MAX449 in combination with Bay11-7082 (Figure 5G). Treatment with MAX449 resulted in a significant reduction in tumor burden (Figure 5H-J). Additionally, flow cytometry results indicated a reduction in CCRL2 and IL-1β expression in PMN-MDSC (Figure 5K-L). However, Bay11-7082 reversed the antitumor activity of MAX449, supporting a critical role for NF-κB signaling suppression in MAX449-mediated tumor control (Figure 5H-L).

### MAX449 significantly enhances the therapeutic outcomes of PD-1 inhibition in various mouse tumor models

The results demonstrated that MAX449 effectively inhibits tumor progression by decreasing the infiltration of PMN-MDSCs. Previous studies have shown that targeting MDSCs in the tumor microenvironment facilitates T-cell activation and improves responses to immunotherapy in mouse models with either sensitivity or resistance to PD-1 blockade 35,47. Consequently, we speculated that MAX449 administration might enhance the effectiveness of PD-1 targeted immunotherapy in cold tumors with intrinsically low responsiveness to PD-1 blockade.

**Figure 6:**
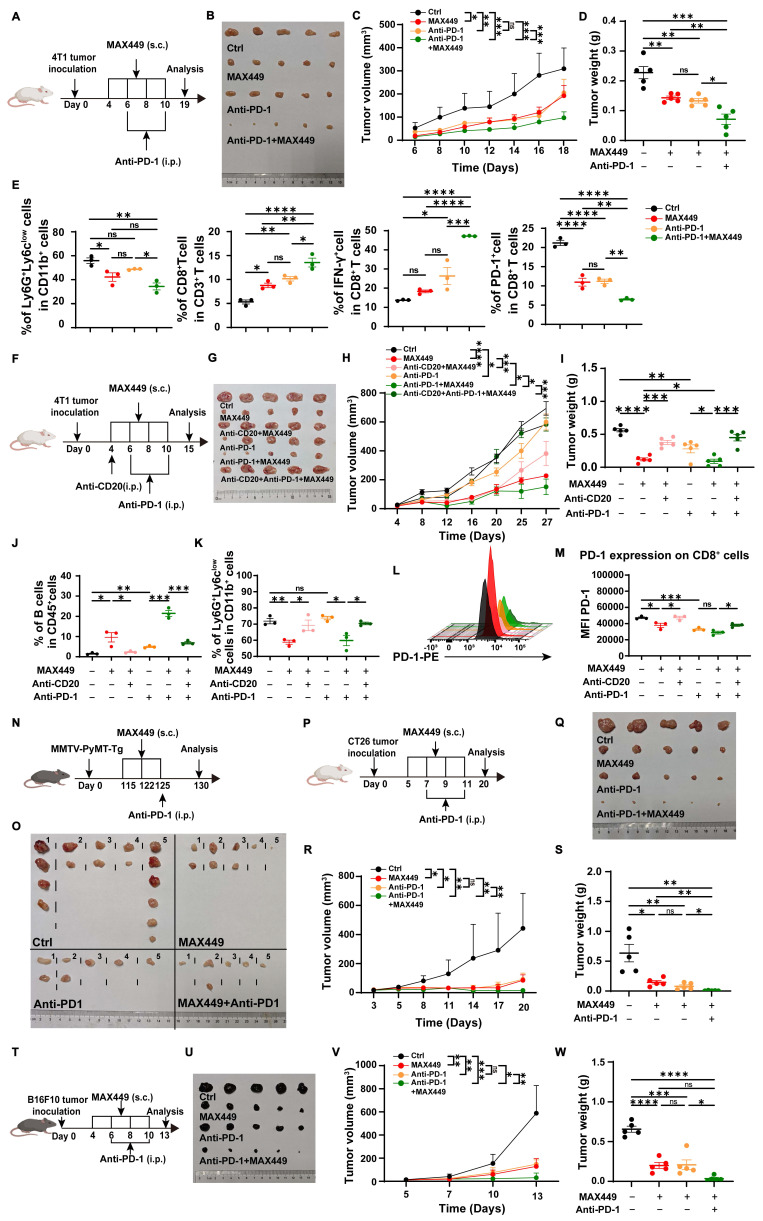
** MAX449 enhanced the antitumor effectiveness of PD-1 antibody on multiple mouse tumor model.** (A) PD-1 antibody was administered to mice bearing 4T1 tumors (100 μg; intraperitoneally, day 4 and 8), MAX449 (0.1 mg; subcutaneously, day 4, 6, 8 and 10) alone or both (n = 5 mice per group). (B) On Day 19, the tumors were collected. The overall growth curves of average tumor volume (C) and tumor weight (D) were compared using a t-test. (E) Flow cytometry analysis of PMN-MDSCs, CD8^+^T, CD8^+^IFN-γ^+^ T cells, and CD8^+^PD-1^+^T cells from mice tumor. (F) Experimental design of Anti-CD20 (250 μg, intraperitoneally, day 4), Anti-PD-1 (100 μg; intraperitoneally, day 6 and 10), and MAX449 (0.1 mg; subcutaneously, day 4, 6, 8 and 10) on 4T-1 tumor transplantation (n = 5 mice per group). (G) On day 15, the tumors were collected. Differences in the growth curves of mean tumor volume (H) and tumor weight (I) were analyzed by t-test. Representative histograms and statistical analyses demonstrate the levels of B cells (J), PMN-MDSC (K), and PD-1 (L-M) on CD8^+^ cells from Ctrl and MAX449 tumor-bearing mice at day-15 post 4T-1 inoculation. (N) A total of 20 MMTV-PyMT transgenic female mice were enrolled in this study and stratified into four experimental group (n = 5 per group). MMTV-PyMT-Tg mice were injected with PD-1 antibody (100 μg; intraperitoneally, day125), MAX449 (0.1 mg; subcutaneously, day 115, 122, and 125) alone or both (n = 5 mice per group). On day 130 after birth, the tumors were collected (O). (P) PD-1 antibody was administered to CT26 tumor-bearing mice (100μg; intraperitoneally, day 7 and 11), MAX449 (0.1 mg; subcutaneously, day 5, 7, 9 and 11) alone or both (n = 5 mice per group). (Q) On Day 20, the tumors were collected. The overall growth curves of average tumor volume (R) and tumor weight (S) were compared using a t-test. (T) PD-1 antibody was administered to B16F10 tumor-bearing mice (100 μg; intraperitoneally, day 6 and 10), MAX449 (0.1 mg; subcutaneously, day 4, 6, 8 and 10) alone or both (n = 5 mice per group). (U) On Day 13, the tumors were collected. A t-test was employed to assess differences across the complete growth curves of mean tumor volume (V) and tumor weight (W). *P* values: **** < 0.0001, *** < 0.001, ** < 0.01, * < 0.05, ns >0.05.

To examine this possibility, we first applied the combination strategy on the cold breast cancer model. In the subcutaneous 4T1 tumor model, we observed that co-treatment with MAX449 and the anti-PD-1 antibody RMP1-14 significantly restrained tumor growth relative to single-agent treatment (Figure 6A-D). A critical mechanism underlying the synergy in immunotherapy is the elimination of PMN-MDSCs alongside the reactivation of CD8^+^ T cells. To characterize MAX449 and/or PD-1 blockade mediated remodeling of the tumor microenvironment, MDSCs and CD8^+^ T cells populations were analyzed in mice bearing 4T1 tumors. Mice were terminated on day 19, prior to full tumor elimination by the combined therapy. Notably, treatment with MAX449 plus PD-1 blockade resulted in a significant reduction in intratumoral PMN-MDSC frequencies relative to control and monotherapy groups. Furthermore, a significant elevation in the proportion of CD8⁺ T cells was observed following the combination treatment. Consistently, among all groups, the MAX449/Anti-PD1 group exhibited the lowest PD-1 expression on CD8⁺ T cells, whereas the highest proportion of IFN-γ-producing CD8⁺ T cells was observed in this group (Figure 6E), indicating an enhanced immunotherapeutic effect. Next, we used a CD20 antibody to deplete B cells before combining MAX449 and PD-1 antibodies, to observe whether the combined anti-tumor effect was diminished. As demonstrated in Figure 6F-M, the combination administration of MAX449 and PD-1 blockade exhibited robust antitumor effects; however, this effect was attenuated by CD20 antibody, further confirming that MAX449 enhances the anti-tumor efficacy of PD-1 antibody through inducing C5a antibodies production.

We also assessed the combination effect in spontaneous adenoma breast tumor models (MMTV-PyMT mouse model) (Figure 6N-O), CT26 (Figure 6P-S), and B16-F10 (Figure 6T-W) xenograft mouse models. Administration of anti-PD-1 treatment on Day 125, when spontaneous tumors were observed, alongside MAX449 (on Days 115, 122, and 125), resulted in a marked suppression of tumor growth by Day 130 (Figure 6N-O). The combination treatment also exhibited superior therapeutic effects compared to PD-1 blockade alone, as evidenced by decreased tumor burden in the CT26 and B16-F10 xenograft mouse models (Figure 6P-W), suggesting a promising potential for combining MAX449 and anti-PD-1 in cancer immunotherapy. In conclusion, MAX449 markedly improves the therapeutic effectiveness of PD-1 antibodies in both cold and hot tumors, offering new strategies to address the lack of response to PD-1 antibodies in cold tumors treatment.

## Discussion

Data from experimental models and clinical studies suggest that the C5a-C5aR pathway is essential for tumor immune evasion and subsequent tumor progression 21. Among various potential mechanisms, inhibition of the C5a-C5aR1 axis may partially alleviate the MDSC-mediated immunosuppressive tumor microenvironment 11,12,39. Given the current landscape in this field, the development of effective, specific, and safe therapeutics targeting the C5a-C5aR pathway is essential. Engineered peptide vaccines capable of inducing sustained polyclonal antibody responses with strong antitumor activity, while inhibiting the C5a-C5aR pathway, constitute a promising therapeutic strategy that offers clear advantages compared with current treatments 24,25.

Here, we advance our checkpoint inhibition strategy by introducing a vaccine targeting C5a. We developed two C5a-based peptide vaccines, MAX449 and MAX450, and proved eliciting strong immunogenic responses. We evaluated the efficacy of the C5a peptide vaccine in vivo using syngeneic mammary tumor models, namely, BALB/c mice bearing 4T1 or CT26 tumors and C57BL/6 mice bearing B16F10 tumors. The MAX449 group exhibited significantly enhanced tumor inhibition. MAX449 inhibited tumor progression by reducing the abundance of PMN-MDSCs while enhancing CD8⁺ T cell infiltration within the tumor microenvironment.

Current research is increasingly focused on identifying synergistic regimens that integrate checkpoint blockade with chemotherapy, radiotherapy, targeted therapy, or additional immunotherapy strategies 8. Although many combination regimens emphasize PD-1/PD-L1 blockade, the immunosuppressive nature of the tumor microenvironment continues to limit therapeutic efficacy 13. Here, we establish a translational framework for clinically evaluating a new combinatorial approach integrating anti-PD-1 antibodies and MAX449, which inhibits C5a-C5aR pathways. Our results demonstrate that MAX449 enhances anti-PD-1 efficacy in both spontaneous adenoma and orthotopic breast cancer models. This approach is based on the premise that targeting immunosuppressive cells in the tumor microenvironment could optimize the activation and expansion of effector T cells. A plausible explanation for the synergistic interaction between MAX449 and anti-PD-1 therapy is that C5a-C5aR signaling drives immunosuppressive mechanisms that limit the anti-tumor efficacy of PD-1 blockade. Supporting the hypothesis, our results indicated that the combination treatment decreased PMN-MDSCs abundance while increasing CD8^+^ T cells frequency, along with reducing PD-1 expression, collectively indicating a more complete restoration of CD8^+^ T-cell effector functions.

In-depth mechanistic studies are essential for accurately assessing the roles of MAX449. SCRNA-seq and flow cytometry analyses indicate that MAX449 reduced the abundance of PMN-MDSCs within the immunosuppressive tumor microenvironment. Additional investigation revealed that MAX449 suppressed PMN-MDSCs migration and immunosuppressive activity by downregulating CCRL2 expression and IL-1β secretion via activation of the NF-κB signaling pathway within the tumor microenvironment. Building on these results, the B cell peptide vaccine MAX449 we developed has the potential to eliminate tumor cells by diminishing PMN-MDSCs migration to the tumor microenvironment and inhibiting their immunosuppressive function, thereby exhibiting anti-tumor activity. This vaccine may offer new insights for the field of cancer therapy.

A central concern when deliberately neutralising an innate-immune mediator such as C5a is the potential for prolonged immunosuppression and increased susceptibility to infection. In this research, we have therefore generated experimental evidence that the anti-C5a vaccine MAX449 can be administered safely in the context of cancer therapy. First, the temporal circulating C5a was characterized in tumour-bearing mice (Supplementary Figure S1A). ELISA analysis revealed that while MAX449 immunization significantly reduced circulating C5a levels compared to controls, the nadir occurred around Day 24 post-initial immunization. Crucially, C5a levels began to recover thereafter, demonstrating that the vaccine-induced suppression is not indefinite and is subject to physiological regulation. The reversibility of the MAX449-induced effect therefore mitigates the risk of sustained complement deficiency while preserving the anti-tumour benefit during the period of active immunisation. Second, we evaluated whether the transient C5a deficiency compromises acute antibacterial defence. On day 24-when C5a concentrations were at their lowest-immunized and control group was challenged intranasally treated with Pseudomonas aeruginosa. Survival curves (Supplementary Figure S1B) proved no statistically significant difference between two groups. These findings indicate that the degree and duration of C5a neutralisation achieved with MAX449 are insufficient to impair the rapid innate immune response required for clearance of a common opportunistic pathogen. Importantly, the infection model employed a Gram-negative organism that is highly dependent on C5a-mediated neutrophil recruitment, thus providing a stringent test of safety 48. In summary, the results substantiate that MAX449 can generate potent anti-tumour immunity while retaining a favourable safety profile.

Besides, the vaccine's safety and specificity as a B-cell epitope hinge on its ability to elicit humoral immunity without inadvertently priming cytotoxic T-lymphocyte (CTL) responses against self-antigens. Our vaccine design purposefully adheres to this principle. Firstly, rational vaccine design focused on B-Cell epitopes: MAX449 incorporates a defined linear B-cell epitope derived from the C5a sequence (amino acids 68-74, KDMQLGR). This epitope was selected on the basis of its location within the native folded C5a protein and its predicted surface accessibility for antibody binding-features that are hallmarks of effective B-cell epitopes 29. This design philosophy has been successfully applied to B-cell vaccines targeting HER-2 and PD-1/PD-L1, where the aim is to mimic conformational or linear structures recognized by B-cell receptors (BCRs) and thereby drive B-cell activation and antibody production 28,49. The core strategy deliberately avoids incorporation of known cytotoxic T-cell (CTL) epitopes. To boost B-cell epitope immunogenicity and promote robust, T-cell-mediated antibody class switching and affinity maturation, we fused the C5a B-cell epitope to a well-characterized, a measles virus fusion protein-derived promiscuous Th epitope (MVF, aa 288-302) and connected by a flexible GPSL linker. This approach is a cornerstone of modern B-cell epitope vaccine design and provides CD4⁺ Th cell help without delivering MHC-class I-restricted CTL epitopes 28. Besides, we also performed an in vitro T-cell activation experiment to verify the absence of C5a-specific CTL activation. Splenocytes obtained from naïve mice were cultured in the presence of MAX449 peptide or left untreated as a control. After 24 h, intracellular interferon-γ production-a hallmark of activated CD8⁺ CTLs-was quantified by flow cytometry 50,51. Results in Supplementary Figure S2 revealed no significant variation in CD8⁺ IFN-γ⁺ T cells percentages between the two groups. These data demonstrate that exposure to MAX449 does not induce CTL activation or IFN-γ secretion under conditions designed to detect such responses. The result is fully consistent with the vaccine's intended B-cell-centric mechanism and the Th-only nature of the MVF epitope.

In the research, we were surprised to find that the anti-CD20 antibody, which alone induces tumor regression, loses its effect when combined with the C5a-targeted vaccine MAX449 (Figure 2F). MAX449 contains the MVF peptide, a promiscuous T cell helper epitope known to expand CD4⁺ T follicular helper (Tfh) cells. Previous research proved that Tfh cells could promote tumor progression by providing help to B cell subsets and shaping an immunosuppressive microenvironment 52,53. Flow cytometric analysis revealed that the proportion of CD4⁺ CXCR5⁺ PD1⁺ Tfh cells was significantly elevated in the MAX449 + anti CD20 group compared with either monotherapy (Supplementary Figure S3). Thus, the antagonism appears to arise from MAX449 driven Tfh expansion, which interferes with anti CD20-mediated anti-tumor effect. The precise mechanisms by which Tfh cells interfere with anti-CD20-mediated B-cell depletion will be explored in future studies.

## Conclusion

**Figure 7:**
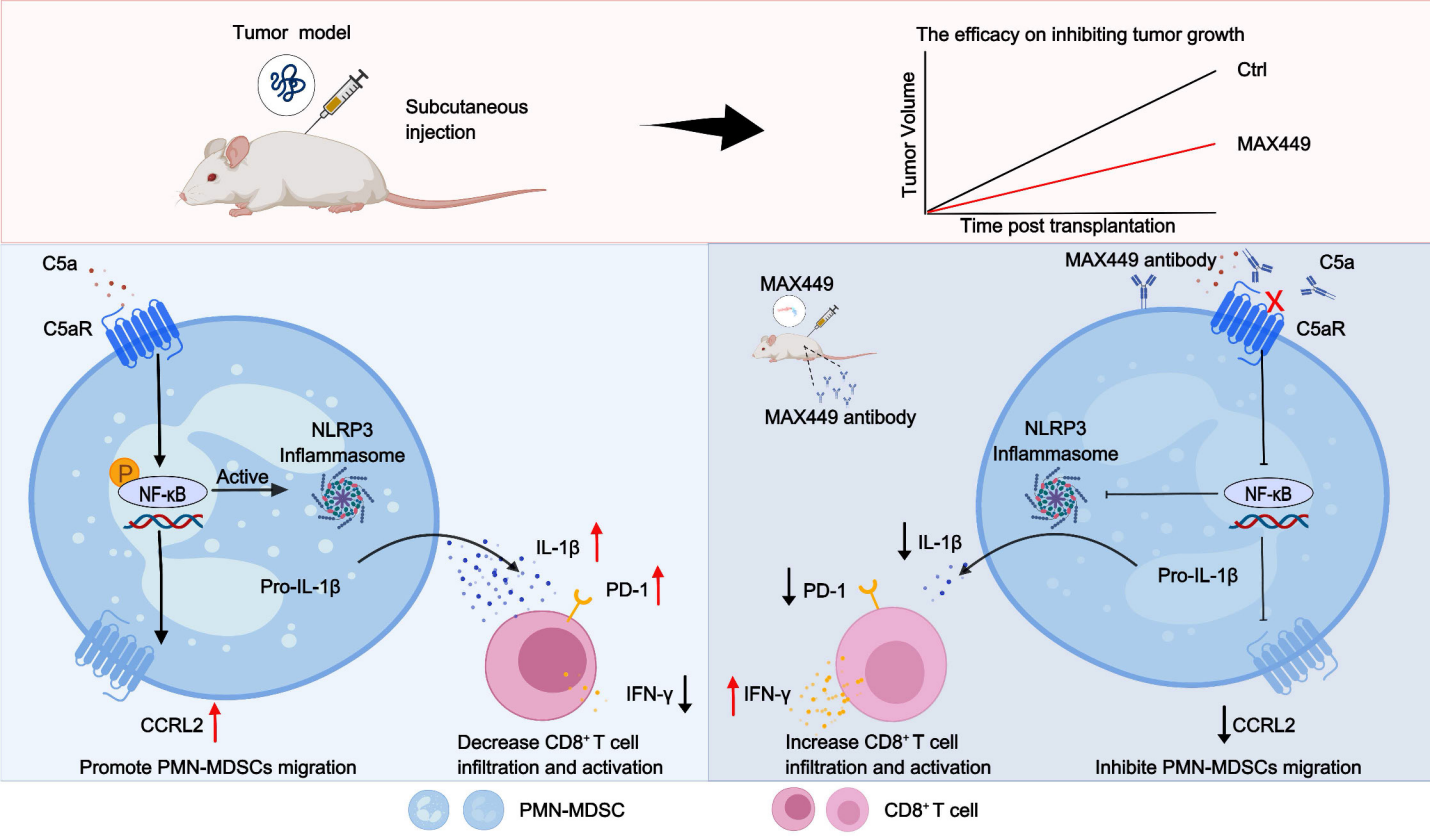
** Schematic of mechanisms that MAX449 exhibits remarkable anti-tumor efficacy against tumors through modulating PMN-MDSCs activity.** MAX449 exhibits exceptional anti-tumor activity. It not only inhibits the migration of PMN-MDSCs to the tumor microenvironment by downregulating CCRL2 expression through the NF-κB signaling pathway but also reduces the immunosuppressive functions of PMN-MDSCs by lowering IL-1β production via the same pathway. Additionally, MAX449 significantly enhances the proportion of CD8^+^ T cells, thereby boosting the antitumor effect.

In summary, we have developed MAX449, a B cell peptide tumor vaccine targeting the C5a-C5aR pathway, which exhibited remarkable anti-tumor effects. This vaccine not only inhibited the migration of PMN-MDSCs to the tumor microenvironment through downregulating CCRL2 expression by the NF-κB signaling pathway but also reduced their immunosuppressive function by decreasing IL-1β production via the same mechanism. Notably, the administration of the vaccine significantly elevates the proportion of CD8^+^ T cells and their antitumor effect. Importantly, it has been demonstrated to strengthen the therapeutic efficacy of PD-1 blockades in mouse models of cold tumors. Overall, MAX449 activates immune system, showcasing the potential of B cell epitope peptide vaccines in anti-tumor therapy. A schematic overview of the entire study workflow is presented in Figure 7.

## Supplementary Material

Supplementary figures.

## Abbreviations

ELISA: Enzyme-linked immunosorben assay
HPLC: High-performance liquid chromatography
scRNA-seq: Single-cell RNA-sequencing
mAbs: Monoclonal antibodies
MDSCs: Myeloid-derived suppressor cells
PMN-MDSCs: Polymorphonuclear myeloid-derived suppressor cells
Treg: Regulatory T cells
FDA: Several Food and Drug Administration
PD-1: Programmed death-1
SDS-PAGE: Sodium dodecyl sulfate-polyacrylamide gel electrophoresis
C5aRA: C5a receptor antagonists
TME: Tumor microenvironment
KEGG: Kyoto Encyclopedia of Genes and Genomes
GO: Gene Ontology

## References

[1] Siegel RL, Miller KD, Wagle NS, Jemal A (2023). Cancer statistics, 2023. CA Cancer J Clin.

[2] Anand U, Dey A, Chandel AKS, Sanyal R, Mishra A, Pandey DK (2023). Cancer chemotherapy and beyond: Current status, drug candidates, associated risks and progress in targeted therapeutics. Genes Dis.

[3] Chandra RA, Keane FK, Voncken FEM, Thomas CR (2021). Contemporary radiotherapy: present and future. Lancet.

[4] He X, Xu C (2020). Immune checkpoint signaling and cancer immunotherapy. Cell Res.

[5] Hegde PS, Chen DS (2020). Top 10 Challenges in Cancer Immunotherapy. Immunity.

[6] Ribas A, Wolchok JD (2018). Cancer immunotherapy using checkpoint blockade. Science.

[7] Twomey JD, Zhang B (2021). Cancer Immunotherapy Update: FDA-Approved Checkpoint Inhibitors and Companion Diagnostics. AAPS J.

[8] Melero I, Berman DM, Aznar MA, Korman AJ, Pérez Gracia JL, Haanen J (2015). Evolving synergistic combinations of targeted immunotherapies to combat cancer. Nat Rev Cancer.

[9] Youn J-I, Nagaraj S, Collazo M, Gabrilovich DI (2008). Subsets of myeloid-derived suppressor cells in tumor-bearing mice. J Immunol.

[10] Mkrtichyan M, Najjar YG, Raulfs EC, Abdalla MY, Samara R, Rotem-Yehudar R (2011). Anti-PD-1 synergizes with cyclophosphamide to induce potent anti-tumor vaccine effects through novel mechanisms. Eur J Immunol.

[11] He J, Chai X, Zhang Q, Wang Y, Wang Y, Yang X (2025). The lactate receptor HCAR1 drives the recruitment of immunosuppressive PMN-MDSCs in colorectal cancer. Nature Immunology.

[12] Theivanthiran B, Yarla N, Haykal T, Nguyen YV, Cao L, Ferreira M (2022). Tumor-intrinsic NLRP3-HSP70-TLR4 axis drives premetastatic niche development and hyperprogression during anti-PD-1 immunotherapy. Sci Transl Med.

[13] Ajona D, Ortiz-Espinosa S, Moreno H, Lozano T, Pajares MJ, Agorreta J (2017). A Combined PD-1/C5a Blockade Synergistically Protects against Lung Cancer Growth and Metastasis. Cancer Discovery.

[14] Senent Y, Tavira B, Pio R, Ajona D (2022). The complement system as a regulator of tumor-promoting activities mediated by myeloid-derived suppressor cells. Cancer letters.

[15] Ortiz-Espinosa S, Morales X, Senent Y, Alignani D, Tavira B, Macaya I (2022). Complement C5a induces the formation of neutrophil extracellular traps by myeloid-derived suppressor cells to promote metastasis. Cancer letters.

[16] Vadrevu SK, Chintala NK, Sharma SK, Sharma P, Cleveland C, Riediger L (2014). Complement c5a receptor facilitates cancer metastasis by altering T-cell responses in the metastatic niche. Cancer Res.

[17] Shu C, Zha H, Long H, Wang X, Yang F, Gao J (2020). C3a-C3aR signaling promotes breast cancer lung metastasis via modulating carcinoma associated fibroblasts. Journal of experimental & clinical cancer research: CR.

[18] Hameed BH, Abdulsatar Al-Rayahi I, Muhsin SS (2022). The Preoperative Serum Levels of the Anaphylatoxins C3a and C5a and Their Association with Clinico-Pathological Factors in Breast Cancer Patients. Archives of Razi Institute.

[19] Chen J, Li GQ, Zhang L, Tang M, Cao X, Xu GL (2018). Complement C5a/C5aR pathway potentiates the pathogenesis of gastric cancer by down-regulating p21 expression. Cancer letters.

[20] Beach C, MacLean D, Majorova D, Melemenidis S, Nambiar DK, Kim RK (2023). Improving radiotherapy in immunosuppressive microenvironments by targeting complement receptor C5aR1. J Clin Invest.

[21] Chen J, Sun ZH, Chen LY, Xu F, Zhao YP, Li GQ (2020). C5aR deficiency attenuates the breast cancer development via the p38/p21 axis. Aging.

[22] Nabizadeh JA, Manthey HD, Panagides N, Steyn FJ, Lee JD, Li XX (2019). C5a receptors C5aR1 and C5aR2 mediate opposing pathologies in a mouse model of melanoma. FASEB journal: official publication of the Federation of American Societies for Experimental Biology.

[23] Xiong J, Kuang X, Lu T, Yu K, Liu X, Zhang Z (2021). C3a and C5a facilitates the metastasis of myeloma cells by activating Nrf2. Cancer gene therapy.

[24] Pio R, Ajona D, Lambris JD (2013). Complement inhibition in cancer therapy. Semin Immunol.

[25] Berraondo P, Minute L, Ajona D, Corrales L, Melero I, Pio R (2016). Innate immune mediators in cancer: between defense and resistance. Immunol Rev.

[26] Carvelli J, Demaria O, Vély F, Batista L, Chouaki Benmansour N, Fares J (2020). Association of COVID-19 inflammation with activation of the C5a-C5aR1 axis. Nature.

[27] McCrary M, Gibbs D, Moreno C, Pollack B 606 Epidermal growth factor modulation of CXCL10 in keratinocytes and cutaneous cancers. Regular and young investigator award abstracts2020. p. A362-A3.

[28] Guo L, Overholser J, Darby H, Ede NJ, Kaumaya PTP (2022). A newly discovered PD-L1 B-cell epitope peptide vaccine (PDL1-Vaxx) exhibits potent immune responses and effective anti-tumor immunity in multiple syngeneic mice models and (synergizes) in combination with a dual HER-2 B-cell vaccine (B-Vaxx). Oncoimmunology.

[29] Landlinger C, Oberleitner L, Gruber P, Noiges B, Yatsyk K, Santic R (2015). Active immunization against complement factor C5a: a new therapeutic approach for Alzheimer's disease. Journal of neuroinflammation.

[30] Ueki I, Abiru N, Kobayashi M, Nakahara M, Ichikawa T, Eguchi K (2011). B cell-targeted therapy with anti-CD20 monoclonal antibody in a mouse model of Graves' hyperthyroidism. Clinical and Experimental Immunology.

[31] Yang H, Deng B, Han X, Wang L, Zhao J, Zhao Y (2025). C5a/C5aR pathway blocking promoted CuS-mediated cancer therapy effect by inhibiting cuproptosis resistance. Journal for ImmunoTherapy of Cancer.

[32] Viola K, Kopf S, Huttary N, Vonach C, Kretschy N, Teichmann M (2012). Bay11-7082 inhibits the disintegration of the lymphendothelial barrier triggered by MCF-7 breast cancer spheroids; the role of ICAM-1 and adhesion. British Journal of Cancer.

[33] Xiao M, Xie L, Cao G, Lei S, Wang P, Wei Z (2022). CD4(+) T-cell epitope-based heterologous prime-boost vaccination potentiates anti-tumor immunity and PD-1/PD-L1 immunotherapy. J Immunother Cancer.

[34] Alshetaiwi H, Pervolarakis N, McIntyre LL, Ma D, Nguyen Q, Rath JA (2020). Defining the emergence of myeloid-derived suppressor cells in breast cancer using single-cell transcriptomics. Science Immunology.

[35] Wang C, Zheng X, Zhang J, Jiang X, Wang J, Li Y (2023). CD300ld on neutrophils is required for tumour-driven immune suppression. Nature.

[36] Luan X, Lei T, Fang J, Liu X, Fu H, Li Y (2024). Blockade of C5a receptor unleashes tumor-associated macrophage antitumor response and enhances CXCL9-dependent CD8(+) T cell activity. Mol Ther.

[37] Li W-H, Su J-Y, Li Y-M (2022). Rational Design of T-Cell- and B-Cell-Based Therapeutic Cancer Vaccines. Acc Chem Res.

[38] Stäubler G, Landlinger C, Mattner F, inventorsVaccine Based on Complement Protein C5a Peptides china patent CN103269711A. 2013-08-28.

[39] Zheng L, Wei J, Li M, Xue C, Wei Q, Wu Z (2025). Understanding the role of C5a/C5aR1-mediated complement activation pathway in tumor progression and therapy resistance. Sci China Life Sci.

[40] Karantanos T, Teodorescu P, Perkins B, Christodoulou I, Esteb C, Varadhan R (2022). The role of the atypical chemokine receptor CCRL2 in myelodysplastic syndrome and secondary acute myeloid leukemia. Sci Adv.

[41] Del Prete A, Martinez-Munoz L, Mazzon C, Toffali L, Sozio F, Za L (2017). The atypical receptor CCRL2 is required for CXCR2-dependent neutrophil recruitment and tissue damage. Blood.

[42] Monnier J, Lewen S, O'Hara E, Huang K, Tu H, Butcher EC (2012). Expression, regulation, and function of atypical chemerin receptor CCRL2 on endothelial cells. J Immunol.

[43] Monteran L, Ershaid N, Scharff Y, Zoabi Y, Sanalla T, Ding Y (2024). Combining TIGIT Blockade with MDSC Inhibition Hinders Breast Cancer Bone Metastasis by Activating Antitumor Immunity. Cancer Discov.

[44] Wang D, Cheng C, Chen X, Wang J, Liu K, Jing N (2023). IL-1beta Is an Androgen-Responsive Target in Macrophages for Immunotherapy of Prostate Cancer. Adv Sci (Weinh).

[45] Lasser SA, Ozbay Kurt FG, Arkhypov I, Utikal J, Umansky V (2024). Myeloid-derived suppressor cells in cancer and cancer therapy. Nat Rev Clin Oncol.

[46] Okamoto M, Liu W, Luo Y, Tanaka A, Cai X, Norris DA (2010). Constitutively active inflammasome in human melanoma cells mediating autoinflammation via caspase-1 processing and secretion of interleukin-1beta. J Biol Chem.

[47] Zhu Y, Chen P, Hu B, Zhong S, Yan K, Wu Y (2024). MDSC-targeting gold nanoparticles enhance PD-1 tumor immunotherapy by inhibiting NLRP3 inflammasomes. Biomaterials.

[48] Pitiot A, Brandus B, Iserentant G, Rolin C, Servais JY, Fouquenet D (2025). Directed-complement killing of Pseudomonas aeruginosa protects against lethal pneumonia. EBioMedicine.

[49] Tobias J, Maglakelidze M, Andric Z, Ryspayeva D, Bulat I, Nikolic I (2024). Phase II Trial of HER-Vaxx, a B-cell Peptide-Based Vaccine, in HER2-Overexpressing Advanced Gastric Cancer Patients Under Platinum-Based Chemotherapy (HERIZON). Clin Cancer Res.

[50] Zhang X, Cheng C, Hou J, Qi X, Wang X, Han P (2019). Distinct contribution of PD-L1 suppression by spatial expression of PD-L1 on tumor and non-tumor cells. Cell Mol Immunol.

[51] Qi X, Li F, Wu Y, Cheng C, Han P, Wang J (2019). Optimization of 4-1BB antibody for cancer immunotherapy by balancing agonistic strength with FcgammaR affinity. Nat Commun.

[52] Mary R, Chalmin F, Accogli T, Bruchard M, Hibos C, Melin J (2022). Hematopoietic Prostaglandin D2 Synthase Controls Tfh/Th2 Communication and Limits Tfh Antitumor Effects. Cancer Immunol Res.

[53] De Monte L, Clemente F, Ruggiero E, Pini R, Ceraolo MG, Schiavo Lena M (2023). Pro-tumor Tfh2 cells induce detrimental IgG4 production and PGE (2)-dependent IgE inhibition in pancreatic cancer. EBioMedicine.

