# Supplementary material for: The novel B-cell epitope peptide vaccine, MAX449, exhibits significant anti-tumor efficacy and enhances the therapeutic effects of PD-1 antibodies on tumors by modulating the activity of PMN-MDSCs
Source: Theranostics. 2026 Jan 14;16(7):3771–89. doi: 10.7150/thno.122439 (PMC12846753; doi:10.7150/thno.122439)
Supplement: Supplementary file 1 — Supplementary figures. [file thnov16p3771s1.pdf]

Supplementary Figures

Supplementary Figure S1

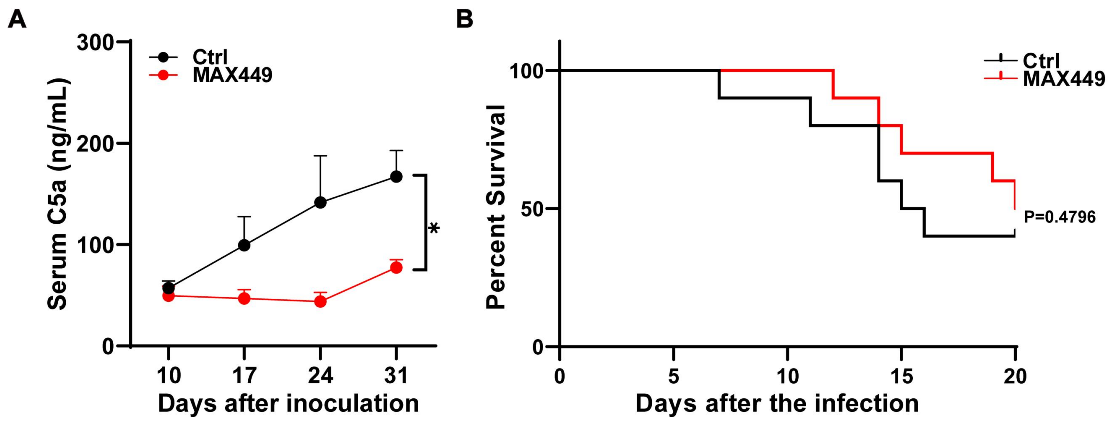

The impact of MAX449 on C5a levels and survival outcomes in mice following infection challenge. (A) C5a concentration in the plasma of Balb/c mice between the Ctrl and MAX449 groups were determined by ELISA assay (n = 3 mice per group). Control group , tumor-bearing mice receiving PBS injection; MAX449 group, tumor-bearing mice treated with MAX449, *P* values: * < 0.05, ns >0.05. (B) A graph illustrating the percentage survival of 4T1 tumor-bearing mice following intravenous challenge with Pseudomonas aeruginosa PAO1 ($1*10^8$ CFU·mL^{-1}, 100 µL per mouse) on day 24 post-tumor inoculation (coinciding with the C5a nadir). Survival was monitored for 20 days after challenge in both control (unimmunized) and MAX449-immunized groups (n = 10 mice per group). *P* value was calculated by log-rank (Mantel-Cox) test using GraphPad Prism 9 software.

Supplementary Figure S2

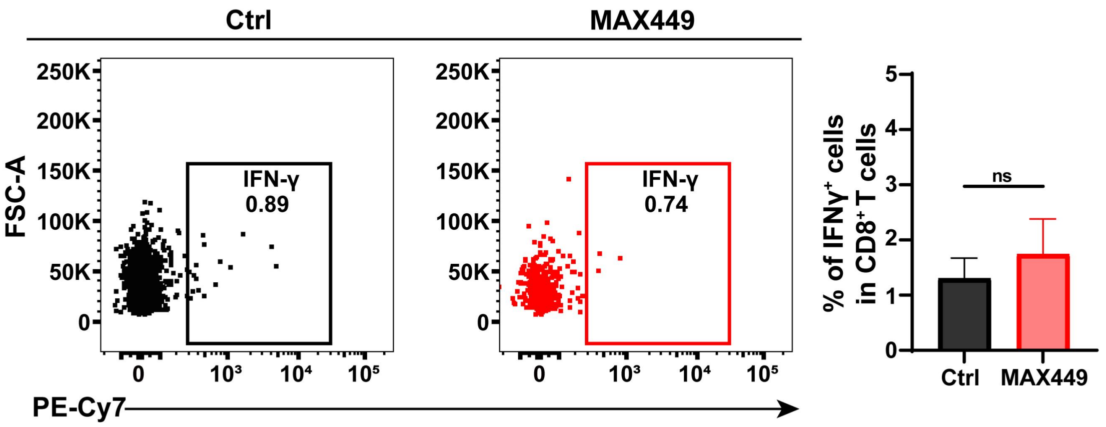

Analyzing cytokines IFN-γ production during CD8^{+}T cell activation by Flow Cytometry Intracellular Staining. Representative flow cytometry scatter plots of % of IFNγ^{+} cells in CD8^{+}T cells (n = 3 mice per group). After the mice had been immunized with MAX449 on four times, splenocytes were harvested and stimulated ex vivo with either PBS (control group) or MAX449 (MAX449 group) for 24 h. *P* values: ns > 0.05.

Supplementary Figure S3

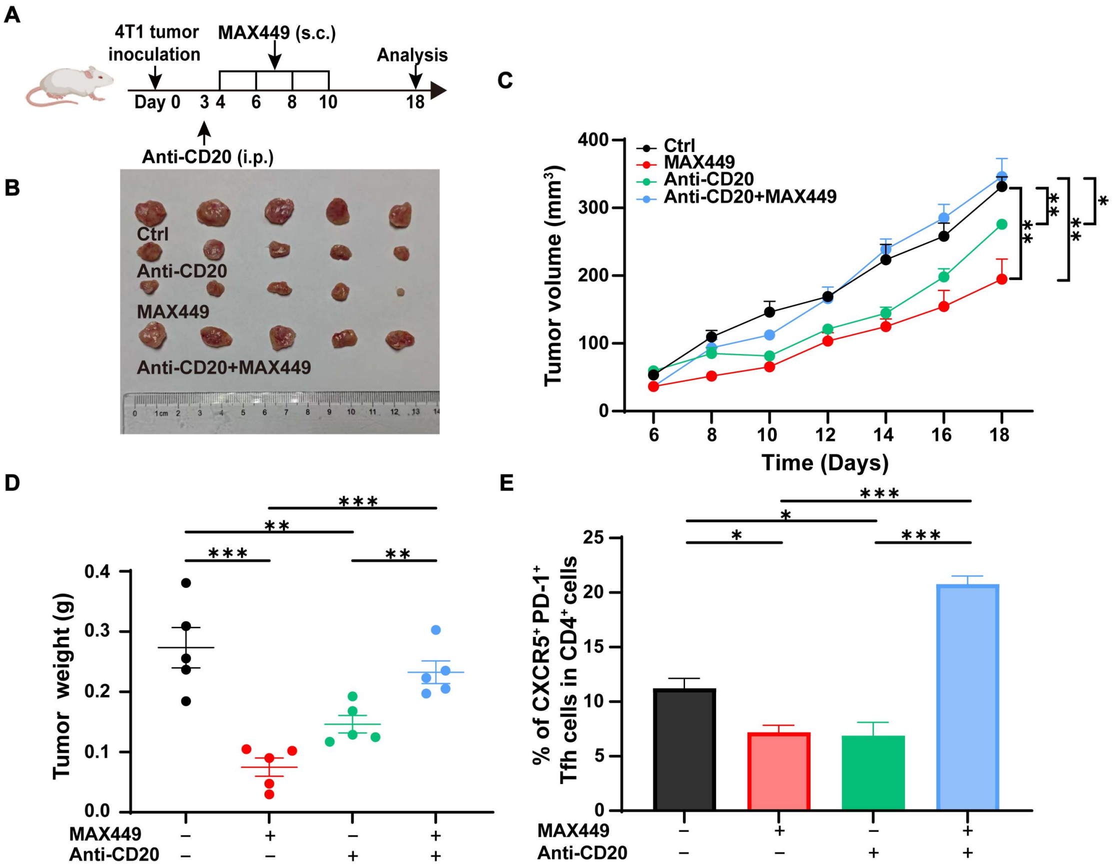

MAX449 blocks C5a/C5aR signaling via B cell-derived anti-C5a antibody. (A) Experimental design of Anti-CD20 and MAX449 on 4T-1 tumor transplantation (n = 5 mice per group). (B) On day 18 after the start of inoculation, the tumors were collected. T-test was used to analyze the whole curves of average tumor volume growing (C) and weight (D). (E) Representative flow cytometry scatter plots of CXCR5$^+$PD-1$^+$Tfh cells. The results are expressed as the Mean ± s.e.m. *P* values: **** < 0.0001, *** < 0.001, ** < 0.01, * < 0.05, ns >0.05.
